# Contribution of the TCRβ Repertoire to Marek’s Disease Genetic Resistance in the Chicken

**DOI:** 10.3390/v15030607

**Published:** 2023-02-22

**Authors:** Cari J. Hearn, Hans H. Cheng

**Affiliations:** Avian Disease and Oncology Laboratory, US National Poultry Research Center, Agricultural Research Service, USDA, East Lansing, MI 48823, USA

**Keywords:** Marek’s disease virus, TCR repertoire, genetic resistance, poultry immunogenetics

## Abstract

Marek’s disease (MD) is a lymphoproliferative disease of chickens induced by Marek’s disease virus (MDV), an oncogenic α-herpesvirus. MDV has increased in virulence, prompting continued efforts in both improved vaccines and enhanced genetic resistance. Model pairs of genetically MD-resistant and MD-susceptible chickens that were either MHC-matched or MHC-congenic allowed characterization of T cell receptor (TCR) repertoires associated with MDV infection. MD-resistant chickens showed higher usage of Vβ-1 TCRs than susceptible chickens in both the CD8 and CD4 subsets in the MHC-matched model, and in the CD8 subset only in the MHC-congenic model, with a shift towards Vβ-1+ CD8 cells during MDV infection. Long and short read sequencing identified divergent TCRβ loci between MHC-matched MD-resistant and MD-susceptible chickens, with MD-resistant chickens having more TCR Vβ1 genes. TCR Vβ1 CDR1 haplotype usage in MD-resistant x MD-susceptible F_1_ birds by RNAseq indicated that the most commonly used CDR1 variant was unique to the MD-susceptible line, suggesting that selection for MD resistance in the MHC-matched model optimized the TCR repertoire away from dominant recognition of one or more B2 haplotype MHC molecules. Finally, TCR downregulation during MDV infection in the MHC-matched model was strongest in the MD-susceptible line, and MDV reactivation downregulated TCR expression in a tumor cell line.

## 1. Introduction

Marek’s disease (MD) is a commonly diagnosed T cell lymphoproliferative disease in chickens, first identified as an infectious polyneuritis by Joseph Marek in 1907 [1]. The causative agent, Marek’s disease virus (MDV) was later identified and characterized as an α-herpesvirus [2]. Since its identification, MDV has been found to cause a series of progressively more severe pathogenic syndromes associated with increasing virulence, likely in response to intensive poultry housing, selection for fast growing chickens, and widespread use of non-sterilizing vaccines [3,4,5,6]. These syndromes include the development of gross lymphoid tumors, neurologic involvement, and acute early mortality [7]. While vaccination strategies have proved largely protective within most flocks, virulence shifts occurring historically every two or three decades [3] have prompted the continued search for alternative control strategies, such as improving host genetic resistance to viral infection and tumorigenesis.

The feasibility of increasing genetic resistance to MD with selection-based methods was demonstrated through the development of a series of highly inbred layer lines of differing MD resistance at Cornell [8] and at our laboratory [9,10]. Early research into genetic based MD resistance focused on the major histocompatibility complex (MHC) [11], as certain MHC (*B* locus) haplotypes were found to exert major effects on resistance to the disease, with the B21 haplotype conferring particularly high resistance [8,10,11,12]. Research into mechanisms of MHC-based resistance has identified differences in the diversity of bound peptides between several MHC class I alleles [13,14]. However, differential genetic resistance to MD is still possible in the context of fixed MHC haplotype, as demonstrated by the Avian Disease and Oncology Laboratory (ADOL) line 6 and line 7 chickens, which are two highly inbred resistant and susceptible layer chicken lines, respectively, with a shared B2 MHC haplotype; these lines do differ at the independently segregating *Rfp-Y* locus, which contains non-classical MHC genes and is not associated with MD resistance in these lines [15]. The difference in disease resistance between lines 6 and 7 indicates that there is additional contributing non-MHC genetic variation to MD resistance which, if characterized, could be used in rational breeding strategies to develop highly MD-resistant chicken lines. One caveat is that prior genetic screens of line 6 and line 7 have failed to identify any major contributing locus beyond the MHC [16], so contributing mechanisms are likely to be polygenic and complex, prompting us to take a broader look at the integrated immune systems of these differentially susceptible lines rather than relying solely on linkage-based genetic screens.

Genomic screens of lines 6 and 7 have been performed at the DNA, transcriptome, and epigenetic levels [17,18,19,20,21,22,23,24]. Not surprisingly, immune genes and immune pathways are frequently identified as being associated with differential response to infection in this model, and differentially expressed genes such as *CD8α*, *IL8*, *CTLA-4*, *IL17A*, and *IL12Rβ2* implicate T cell transcriptional pathways in MD resistance [25]. Recently, transcriptomics work in our laboratory has identified many candidate resistance genes and pathways that are differentially expressed (DE) or regulated (showing allele-specific expression, ASE) by MDV infection in lines 6 and 7 [26,27,28]. Multiple immune pathways, including innate (TLRs, apoptosis), NK cell and cytokine signaling (JAK-STAT) pathways have been identified using these techniques. In addition, studies of local cytokine expression in the spleens of MDV-infected birds have indicated that susceptible line 7 birds primarily upregulate genes in the T-regulatory and Th-2 response paradigms, while line 6 birds have a more robust Th-1 component to their immune response [29].

Early studies on the functionality of T cells in MD-resistant and susceptible lines focused on bulk lymphocyte responses to nonspecific mitogens, such as lectins [30,31,32]. Differential T cell proliferation capacity was offered as a potential source of variability in either cellular immunity or oncogenic transformation. However, results are conflicting between the studies, with whole blood proliferation assays suggesting a consistently higher ConA mitogen response in the susceptible line 7 even when very different T cell counts are taken into consideration [30] while purified lymphocyte assays indicate that line 6 and line 7 do not differ in PHA mitogen response [31]. Functional assays of specific T cell subsets were not performed, as these studies occurred prior to the development of T cell marker-specific antibodies.

Increasing knowledge about the immunobiology of non-mammalian species, including avians, has allowed us to begin characterizing the T cell responses of these species to important pathogens. Functionally, the chicken T cell receptor (TCR) system appears to overlap, at least broadly, with mammalian immunity, with homologous TCR complex components, including TCR heterodimers, CD3 chains, and CD4 and CD8 co-receptors [33,34,35,36,37]. Importantly, the avian immune system is characterized by reductions in the size of antigen receptor multi-gene blocks, including MHC, TCR and immunoglobulin loci, possibly as an adaptation to flight [38,39]; while recombinational diversity is preserved in B and T cells through VDJ recombination, pre-existing diversity in MHC loci and the TCR and B cell receptor (BCR) genes available for recombination are reduced, which may allow larger selection effects to occur in the immune receptors of birds, and precipitate an “arms race” between avian pathogens and their hosts [39]. For example, the MHC locus only encodes two classical MHC genes each of class I and class II (reviewed in [12]), and these appear to be co-evolving with antigen processing machinery in order to maximize utility for response to specific pathogens [39,40,41,42,43]. Similarly, the chicken *TCRβ* locus, located on chromosome 1, includes approximately 10 variable genes (less than a one-third that of a mouse), and these can be categorized into two or three families of closely related genes [44,45]. Recent studies characterizing the reference chicken (Red jungle fowl) *TCRβ* locus have produced disparate results [45,46], which are likely due to differences in sequencing and assembly methods, and highlight the challenges associated with sequencing across highly-repetitive immune loci.

Here, we show that the *TCRβ* repertoire of MD-resistant line 6 chickens and MD-susceptible line 7 chickens are divergent, and that this divergence correlates with differences in CD8+ T cell responses, but not CD4+ T cell responses, in vivo. Additionally, we demonstrate that MHC haplotypes that show differential MD resistance also induce differences in the *TCRβ* repertoire of CD8+ T cells. Together, these findings suggest that the streamlined avian TCR system can be optimized either for or against resistance to pathogens such as MDV, and, due to its reduced size, may be more susceptible to significant changes induced by natural or artificial selection.

## 2. Materials and Methods

### 2.1. Animals and Viruses

All animal studies were performed according to ADOL Institutional Care and Use Committee (IACUC) guidelines following approved Animal Use Protocols (approval numbers #2015.09, #2016.11, #2016.13, #2017.04, #2018.01, and #2021.07.) SPF chicken lines used in this study included ADOL lines 6 and 7, which are, respectively, MD-resistant and MD-susceptible B2 haplotype inbred layers [9]; ADOL MHC congenic layer lines 15I_5_.B21 and 15I_5_.B19, which are congenic for the MD-resistant B21 haplotype and the likely partially recombinant MD-susceptible B19^15-P^ [10,47] haplotype, respectively; and ADOL 6C.7 recombinant congenic layer strains derived from ADOL lines 6 and 7 [10].

For all challenge experiments, mixed-sex chicks were routinely placed in Horsfall-Bauer isolators at 1 day post hatch and given feed and water ad libitum throughout the experiment; moribund birds were removed and humanely euthanized within 24 h.

Experiment 1: For replicate 1, chicks from ADOL lines 6 and 7 [9] were inoculated at 1 day post hatch with 2000 pfu Md5 strain MDV (very virulent pathotype), in vitro passage no. 7, by intra-abdominal injection. For replicate 2, Md5 in vitro passage no. 8 was used. Birds were humanely euthanized at the specified collection days and spleens were collected for flow cytometry. Spleens from 3 chicks were pooled together for each sample at early ages (0 and 5 days), while individual chick spleens were assayed at later ages. For sampling in replicate 1, *n* = 4 sample pools of 3 birds each per group at day 5; *n* = 4 birds per group at days 8, 14, 21 and 28; and *n* = 6 remaining birds per control group at day 35. For replicate 2, *n* = 3 sample pools of 3 birds per group at each timepoint from days 0 and 5, with the exception of 2 sample pools of 3 birds in the day 5, line 7 uninfected group; *n* = 5 birds per group from days 8, 14, and 21; and *n* = 5 and 8 remaining birds per control group for lines 6 and 7, respectively, at day 35.

Experiment 2: A single replicate was conducted similarly to experiment 1; however, chicks from ADOL MHC congenic lines 15I_5_.B19 and 15I_5_.B21 were used for this experiment [10]. The inoculum was 2000 pfu JM/102W strain MDV (virulent pathotype), in vitro passage no. 14, given at 1 day post hatch by intra-abdominal injection. For experiment 2, spleens from individual chicks were assayed at each timepoint: *n* = 4 birds per group at each timepoint (days 5, 8, 14, 21 and 28).

Experiment 3: Chicks from ADOL lines 6 and 7 were inoculated with 500 pfu Md5 strain MDV, in vitro passage no. 8, at day of hatch by intra-abdominal injection. Splenic tissues were collected from individual birds humanely euthanized at 7 days of age for flow cytometric analysis. *n* = 6 birds per group in a single replicate.

### 2.2. Spleen and Peripheral Blood Mononuclear Cell Immunophenotyping (Lines 6 and 7)

Experiments 1 and 2: Spleens were cut to open the splenic capsule and up to ~250 μL volume of tissue was gently homogenized with a Teflon-coated pestle to single-cell suspension in 250 μL LM media (50% Lebowitz, 50% McCoy’s 5A) in 2 mL tubes and counted. For experiment 2, peripheral blood mononuclear cells (PBMCs) were also isolated from whole blood over a Histopaque^®^-1077 gradient according to the manufacturer’s instructions (product #10771, MilliporeSigma, Burlington, MA, USA). For each sample, ~1 million cells were immunolabeled with the following antibody panel: CD3-Alexa Fluor™ 700 (clone CT-3, Southern Biotech, Birmingham, AL, USA); CD8α-FITC (clone 11–39, Bio-Rad Laboratories, Hercules, CA, USA); CD8β-APC (clone EP42, Novus Biologicals, Centennial, CO, USA; experiment 1, replicate 1) or CD4-Pacific Blue (clone CT-4, Southern Biotech; experiment 1, replicate 2 and experiment 2); TCRαβVβ1-PE (clone TCR2, Southern Biotech); TCRαβVβ2-SPRD (clone TCR3, Southern Biotech); and TCRγδ-biotin (clone TCR1, Southern Biotech; experiment 1, replicate 1, days 5–14 only). Immunolabeling was performed in 100 μL of PBS with 1% FBS (FACS buffer) at 4 °C for 30 min in the dark. For experiment 1, replicate 1, days 5–14 only, samples were washed once in 200 μL of FACS buffer and resuspended in 100 μL of FACS buffer for secondary staining with streptavidin-Brilliant Violet 421 (Thermo Fisher Scientific, Waltham, MA, USA). Samples were spun down and then washed two times in 200 μL of FACS buffer and re-suspended in FACS buffer for analysis. In experiment 1, replicate 2 and experiment 2, live-dead discrimination was performed with DAPI (MilliporeSigma) at 0.005 μg/mL. Flow cytometry was performed on a BD Influx™ machine (Becton, Dickinson & Company, Franklin Lakes, NJ, USA). Data were analyzed in FlowJo™ versions 9 and 10 (Becton, Dickinson & Company). Flow gating was performed for scatter characteristics (FSC x SSC), viability (DAPI negative; experiment 1, replicate 2 and experiment 2 only), and positive CD3 expression before subgating into CD8α+ CD8β+ (experiment 1, replicate 1) or CD4+ CD8α- and CD4- CD8α+ populations (experiment 1, replicate 2, and experiment 2). Each T cell population was further subgated into TCR2+ TCR3- and TCR2- TCR3+ populations, respectively; TCR2+ TCR32+ cells were infrequent and are excluded from these analyses.

Experiment 3: Spleens were homogenized in LM media as in experiments 1 and 2, washed and cryopreserved in freezing medium containing 10% DMSO and 17% FBS at −80 °C for later analysis. Frozen samples were thawed quickly (less than 1 min) on a 95 °C heat block to prevent macrophage lysis. Thawed samples were washed in LM media, strained through wire mesh and then filtered through Falcon™ 35 μm cell-strainer caps (product #352235, Corning, Corning, NY, USA) to remove aggregates. One million cells per sample were immunolabeled with each of 2 antibody panels: Monocyte/macrophage-Alexa Fluor™ 647 (clone KULO1) and Bu-1-Pacific Blue (clone AV20) and either MHCI-FITC (clone F21-2; panel 1) or MHCII-FITC (clone 2G11; panel 2). All antibodies were obtained from Southern Biotech, Inc. Immunolabeling was performed as in experiments 1 and 2; samples were kept on ice at all steps. Flow cytometry was performed on a Novocyte cytometer (Agilent Technologies, Santa Clara, CA, USA), and analysis was performed in FlowJo™ v. 10; samples were singlet-gated, gated for mononuclear cells on FSC/SSC, and population-gated into KULO1+ and Bu-1+ populations before measurement of MHCI or MHCII expression as median fluorescent intensity (MFI). To minimize sample processing time, samples were processed in batches containing 3 samples from each group, and MFIs were normalized to the batch average (nMFI) prior to statistical analysis. One sample was removed from analysis in the line 7 uninfected group due to poor cell recovery. nMFI for each sample was calculated as:

nMFI = MFI_sample_/([Σ MFI_samples in batch_/N_samples in batch_])

### 2.3. PBMC Immunophenotyping (Recombinant Congenic Strains)

Blood samples were obtained from 2 adult colony-cage-housed breeding hens from each of 18 different 6C.7 recombinant congenic strains [10], as well as 1 hen from each parental line. PBMCs were isolated by gradient centrifugation over Histopaque^®^-1077. For lines A–D, 1 million PBMCs were immunolabeled with CD8α-FITC (clone 11–39, BioRad Laboratories, Hercules, CA, USA), CD4-APC (clone CT-4, Novus Biologicals), TCRαβVβ1-PE (clone TCR2, Southern Biotech) and TCRαβVβ2-SPRD (clone TCR3, Southern Biotech); immunolabeling was performed as in experiments 1–3; and flow cytometry performed on a BD FacsCalibur™ machine (Becton, Dickinson & Company). For all additional lines, immunolabeling with TCRαβVβ1-PE and TCRαβVβ2-SPRD only was performed. Data were analyzed in FlowJo™, v. 10. Flow gating for scatter characteristics (FCS x SSC) was performed, and gated cells were subgated for CD4+ CD8α- or CD4- CD8α+ populations, or TCR+ (TCR2+ OR TCR3+) populations, respectively; and each population was further subgated into TCR2+ TCR3- and TCR2- TCR3+ subsets.

### 2.4. Reactivation Assays

UAO4 cells [48] were routinely cultured in RPMI-1640 (product #30-2001, American Type Culture Collection, Manassas, VA, USA) with 20% FBS, penicillin (100 IU/mL, streptomycin (100 μg/mL), and 1× Gibco™ GlutaMAX™ (product #35050079, Thermo Fisher Scientific). For reactivation assays, cells were cultured at 1 × 10^6^ cells/mL in the same media containing 10 μg/mL 5-bromo-2′-deoxyuridine (BrdU; product #B5002, MilliporeSigma), or unlabeled anti-CD3 (clone CT-3, Southern Biotech) and unlabeled anti-CD28 (clone AV7, Southern Biotech) antibodies at diluted equal concentrations ranging from 1:250 to 1:1000. Assays were set up in 48-well tissue-culture plate format with 0.5 mLs per plate volume (5 × 10^5^ total cells per well). Additional samples were cultured in wells that were pre-coated with unlabeled anti-TCRαβVβ1 (clone TCR2, Southern Biotech) antibody at 1:100, 1:200 or 1:1000 dilution in PBS and rinsed with PBS prior to plating. At 48 h, a 3/4ths-volume media replacement was performed for controls, BrdU, and anti-TCRαβVβ1 samples to maintain growth; BrdU was included in the replacement media for BrdU-treated samples. At 72 h, samples were washed in PBS and labeled for flow cytometry with anti-TCRαβVβ1-PE (clone TCR2; Southern Biotech) or anti-MHC class II-PE (clone 2G11; Southern Biotech). One million cells per sample were immunolabeled as in experiments 1–3. Samples were analyzed by flow cytometry on a BD FacsCalibur™ machine prior to and after immunolabeling. Flow gating was performed for scatter characteristics (FSC x SSC), subgated by GFP expression, and TCRαβVβ1 expression (mean fluorescence intensity), and MHC class II expression (mean fluorescence intensity) was measured for GFP+ and GFP- subpopulations. Viability studies were performed separately with propidium iodide staining.

### 2.5. Reactivation-Inhibition Assay

This assay was performed similarly to the reactivation assays except that cells were pre-incubated for 1 h with 20 μM, 50 μM, or 100 μM of the caspase inhibitor compound Z-VAD-FMK (product #sc-3067, Santa Cruz Biotechnology, Dallas, TX, USA), or an equivalent volume of vehicle (2 μL/mL, 5 μL/mL, or 10 μL/mL DMSO) prior to addition of either 40 μg/mL BrdU, or unlabeled anti-CD3/CD28 antibodies at 1:500 dilution. Z-VAD-FMK and BrdU were maintained in the media throughout the 72-h culture with ½ volume media replacement of all samples at 48 h; additional anti-CD3/CD28 antibodies were not added during media replacement. Immunolabeling of 1 million cells per sample was performed as in reactivation assays with the same antibody panel.

### 2.6. Proliferation Assays

Uninfected line 6 and line 7 mixed-sex chicks were housed as in experiments 1–3, and humanely euthanized for spleen collection at the specified timepoints. Sample pooling was performed as follows due to the small size of chick spleens. Spleens from birds 5 days old or less were pooled in groups of 4; samples from birds 15 days old and 22 days old were pooled in groups of two; and spleens from birds at days 26 and 36 days of age were assayed individually. Spleens/pools were assayed for proliferation with concanavalin A (ConA; product #C0412, MilliporeSigma), phytohaemagglutanin (PHA; product #L9017, MilliporeSigma), or both, by the following method, depending on sample processing yields; sample numbers at each timepoint are summarized in Supplementary Table S1. Spleens were cut to open the splenic capsule, pooled and gently homogenized in 250 μL of LM media in 2-mL tubes with a Teflon-coated pestle (5 days of age or less) or in 2–4 mLs of LM media on petri dishes with the plunger of a syringe (older than 5 days). Spleen lymphocytes were purified by gradient centrifugation over Histopaque^®^-1077 and labeled with a fluorescent tracking dye (Invitrogen™ CellTrace™ Far Red; product #C34564, Thermo Fisher Scientific); cells were counted and CellTrace labeling was performed following kit instructions with 5 × 10^7^ cells and 0.5 μL of 1 mM CellTrace Far Red stock solution per mL of staining volume in PBS and 20 min incubation time at 37 °C in the dark, followed by quenching for 5 min in the dark at 37 °C with 5 volumes of culture medium (see following). Two million cells/sample were cultured in 2 mL of IMDM (product #30-2005, American Type Culture Collection) with 8% FBS, 2% heat-inactivated chicken serum, plus penicillin/streptomycin. Additionally, 10 μg/mL ConA or 100 μg/mL PHA were included in the media to stimulate proliferation, and samples were incubated at 41 °C in 5% CO_2_ for 72 h. Assays were set up with 3 technical replicates when possible (depending on sample yields), and technical replicates of the same sample were combined after incubation and prior to immunolabeling. Cells were counted and 1 million cells each were labeled separately with anti-CD4-FITC (clone CT-4, Southern Biotech) or anti-CD8α-FITC (clone 11–39, BioRad Laboratories). Immunolabeled samples were stained for viability with 1 μM Invitrogen™ Sytox™ Blue Dead Cell Stain (Thermo Fisher Scientific) and analyzed on a BD Influx™ machine, or with 0.1 μg/mL propidium iodide (MilliporeSigma) and analyzed on a BD FacsCalibur™ machine; and proliferation indices were analyzed in FlowJo v.9. A control sample from each CellTrace Far Red-labeled splenocyte sample was fixed at the time of labeling in 4% paraformaldehyde for 10 min, washed in PBS and stored at 4 °C until the flow cytometry run. Flow gating was performed for scatter characteristics (FSC x SSC), viability (Sytox Blue for assays run on the Influx; propidium iodide for assays run on the FacsCalibur) and gated cells were subgated into CD4+ or CD8+ populations. Proliferation indices based on Cell Trace Far Red fluorescence were modeled separately for each sample, with 7–9 total peaks modeled for best fit starting from the initial peak 0 lymphocyte fluorescence in the matched fixed, stored CellTrace-labeled controls. Proliferation index for CD4+ and CD8+ cells is reported (referred to as expansion index in FlowJo v. 9 [49]) and represents the fold-expansion of the gated population.

### 2.7. Cell Cycle Analysis Assays

Uninfected line 6 and line 7 mixed-sex chicks were housed as in experiments 1–3, and humanely euthanized for spleen collection at the specified timepoints. Spleens from 15-day-old birds were pooled in groups of 2; spleens from 29-day-old birds were assayed individually; *n* = 6 pools or individuals per group at each timepoint. Spleens were homogenized as in proliferation assays. Splenic lymphocytes were purified by two rounds of gradient centrifugation over Histopaque-1077 (MilliporeSigma) and further cultured for 48 h with 10 μg/mL ConA or 100 μg/mL PHA as in the proliferation assays, then pulsed for 1 h with 1 μL 5-ethynyl-2′-deoxyuridine (EdU)/2 mL sample using the Invitrogen™ Click-IT™ EdU Alexa Fluor™ 647 Kit (product #C10424, Thermo Fisher Scientific) and click-labeled for EdU detection according to the manufacturer’s protocol. Cells were immunolabeled with anti-CD4-FITC (clone CT-4, Southern Biotech) and anti-CD8α-PE (clone CT-8, Southern Biotech) antibodies subsequent to click-labeling and analyzed on a BD FacsCalibur machine. Flow cytometry data were analyzed for percent S-phase in Flowjo, v.10. Flow gating was performed for scatter characteristics (FSC x SSC), and gated cells were subgated into CD4+ CD8α- and CD4- CD8α+ populations. Percent S-phase was calculated as the proportion of Edu+ cells within each population.

### 2.8. Spectratyping Assay

Spleens from line 6 and 7 chicks uninfected or infected with 2000 pfu Md5 strain MDV at day of hatch, collected in the course of a previous study [50] at 7, 14 and 21 days post-infection, were spectratyped for *TCRβ* length. cDNA was prepared from spleen samples preserved at −20 °C in RNAlater, using the Absolutely RNA Miniprep kit (product #400800, Agilent Technologies) followed by the Invitrogen™ Superscript™ First Strand Synthesis kit (product #11904018, Thermo Fisher Scientific) or Applied Biosystems™ High Capacity cDNA kit (product #4368814, Thermo Fisher Scientific) and oligo-dT primers. Nested PCR was performed for TCR Vβ1 and TCR Vβ2 using previously described primers and methods [51]. PCR products were diluted at 1:200 and fragment analysis was performed on an ABI (Applied Biosystems™,., Thermo Fisher Scientific) 3730xL machine. Fragment data were analyzed in Peak Scanner v.2 (Thermo Fisher Scientific).

### 2.9. DNA Sequence Analysis

Pooled whole blood samples were previously collected, extracted for DNA, and sequenced from 7 male birds each of lines 6 and 7 in follow-up efforts to [52]; DNA extractions were performed using the Qiagen QiaAmp DNA Blood kit (Qiagen, Hilden, Germany), and Illumina libraries were prepared and sequenced by 100-bp Illumina sequencing through DNA Landmarks, Inc. (Saint-Jean-sur-Richelieu, QC, Canada). Sequence data were submitted to the NCBI Sequence Read Database (Bioproject: PRJNA574415). Sequence data were aligned in BWA-MEM v. 0.7.17 [53] against the Gallus_gallus-5 reference genome [54] with default parameters, de-duplicated, and locally realigned. Vβ1 regions were identified within the reference sequence by BLAT search using published and unpublished annotated chicken *TCRβ* mRNAs present in Genbank (Genbank M37801-M37805 [55]; Genbank EF554742-EF554782: Xia, Yang and Zhang, direct submission in 2008). Mapped reads which overlapped these regions were realigned to a reference sequence for Vβ1 (Genbank EF55473.1/ABU93628.1) in IGV v. 2.4.5 [56,57], and variants were called using Freebayes v. 1.2.0 [58], using 3-bp and 10-bp haplotype windows. Long-window haplotypes within the CDR1 region were identified manually in IGV.

### 2.10. Vβ1 CDR1 Haplotyping of RNA Sequence Data

Illumina RNA-seq data generated from a previously published study [28] were analyzed for TCR Vβ1 usage. Briefly, line 6 × 7 F_1_ chicks were infected at 2 weeks post hatch with 2000 pfu Md5 strain MDV or uninfected. At 4 days post-infection, spleen samples were collected, RNA extracted and 100 bp paired-end Illumina HiSeq RNA-seq was performed [28]. Raw RNA-seq data were uploaded to the NCBI Sequence Read Database (BioProject: PRJNA574432). RNA-seq data were quality-checked with FastQC v. 0.11.7 [59], reads were trimmed to 90 bp with FastX-trimmer v. 0.0.14 [60], and aligned to the Gallus_gallus-5 reference genome with BWA-MEM v. 0.7.17 [53]. Alignment files were converted to BAM format, sorted and indexed, and reads mapping to chr1:78,000,000–78,300,000 (a region spanning the *TCRβ* locus) were obtained using Samtools v. 1.9 [61]. The resulting truncated alignment files were converted to fastq sequences with Samtools, re-aligned in BWA against a reference TCR Vβ1 sequence [Genbank EF55473.1/ABU93628.1], and mapped reads from pair mates 1 and 2 were merged into a single alignment file using Samtools. TCR Vβ1-mapping alignments from 7 infected and 7 uninfected birds were analyzed together in Freebayes to identify CDR1 haplotypes, using the following command line flags:

[--haplotype-length 40 -r <vb1_sequence>:190-240 -C 4 --pooled-continuous]

Which allows haplotype construction of up to 40 bp in length, limits the search to a 50-bp region spanning the CDR1 mutational hotspot, requires a minimum of 4 calls per haplotype, and treats the sample ploidy as unknown.

### 2.11. CDR3 Identification in RNA Sequence Data

A subset of three MDV-infected and uninfected samples each from the above line 6 × 7 F_1_ Illumina RNAseq dataset were analyzed for the presence of CDR3 sequences. The trimmed fastq files were searched against a set of conserved Vβ1 primer sequences (Supplementary Table S1; identified across Genbank submissions of chicken *TCRβ* mRNAs; see Section 2.9 above) adjacent to the 5′ end of the chicken CDR3 region, in both forward and reverse-complement orientations, using fastq-grep v. 0.8 [62]. Identical reads were collapsed to remove duplicates with FastX-Collapser v.0.0.14 [60]. Reads in the reverse orientation were reverse-complemented with FastX-Reverse-Complement v.0.0.14 [60], and the combined, single-orientation CDR3-adjacent reads from both mate pairs per sample were aligned with Clustal Omega v. 1.2.2 [63], and the alignments manually realigned and translated in Geneious Prime^®^ v. 2022.2.1 (https://www.geneious.com, accessed on 3 March 2022).

### 2.12. Protein Sequence Analysis

A Vβ1 protein structure was modeled for the chicken *TCRβ* reference sequence published in Genbank (ABU93628.1; contains Vβ1 and Jβ4.2 segments; residues 17–257 were modelled), using default SWISS-MODEL parameters [64,65] with a crystallography-supported mouse TCR-pMHC structure as the template (PDB ID 3mbe.1.E) [66]. Substitutions were made in the Vβ segment and re-modelled against the 3mbe.1.E template, followed by structural comparison between models in Raptor-X [67,68].

### 2.13. PacBio Long Read DNA Sequencing

Whole blood samples from one healthy adult male bird each of lines 6 and 7 were extracted for DNA using a non-column method, which involved overnight lysis in SDS/TE buffer (10 mM Tris, 1 mM EDTA, 0.5% SDS) with proteinase K (60 μg/mL), protein precipitation with Qiagen Precipitation Solution and ethanol precipitation of the soluble DNA phase to minimize shearing. DNA samples were resuspended in distilled water and submitted for PacBio Sequel sequencing by the USDA-ARS Genomics and Bioinformatics Unit (Stoneville, MS). Raw sequences were submitted to the NCBI Sequence Read Archive (BioProject: PRJNA574234). Read correction, overlapping and de novo assembly was performed using Canu v. 1.8 [69], and assemblies were examined for contig continuity using Blast+ v. 2.7.1 [70] and Bandage v. 0.8.1 [71]. Assembly annotations were performed in Geneious Prime v. 2022.2.1 and alignment of TCR Vβ sequences was performed with Clustal Omega v.1.2.2 [63].

### 2.14. Statistics

Ex vivo flow cytometry data (from day 5 post-infection to day 35) were analyzed by three-way analysis of variance in Jamovi 0.8.0.10 (www.jamovi.org, accessed on 13 October 2017), followed by post-hoc Tukey’s tests for significance. In vitro flow cytometry data were analyzed by Bonferonni-corrected Student’s *t*-testing in Prism 8.0.0 for MacOS (Graphpad Software, San Diego, CA, USA; http://www.graphpad.com, accessed on 10 October 2018; proliferation assays) or Excel for Mac v. 15.41 (Microsoft Corporation, Redmond, WA, USA; cell cycle analysis assays), or by 2-way ANOVA with Sidak’s multiple comparisons tests in Prism 8.0.0 for MacOS (reactivation assays). Plots were generated in Prism 8.0.0 for MacOS) or Excel for Mac v. 15.41. TCR spectratyping data were analyzed for total divergence score [72], using the following formula:

$$\mathrm{Total}\;\mathrm{Divergence}\;\mathrm{Score}=\Sigma (|x_{\mathrm{peak}\;\mathrm{fractional}\;\mathrm{area}}-{\bar{x}}_{c\;\mathrm{peak}\;\mathrm{fractional}\;\mathrm{area}}|)$$

where average control peak fractional areas are calculated across all uninfected controls of the same chicken line at the same timepoint (or, for individual control birds, all remaining uninfected controls of the same line at that timepoint).

## 3. Results

### 3.1. In Vitro Splenic T Cell Proliferation in Response to Mitogens Are Similar between MD-Resistant and MD-Susceptible Chickens with the Same MHC B2 Haplotype until 36 Days of Age 

**Figure 1:**
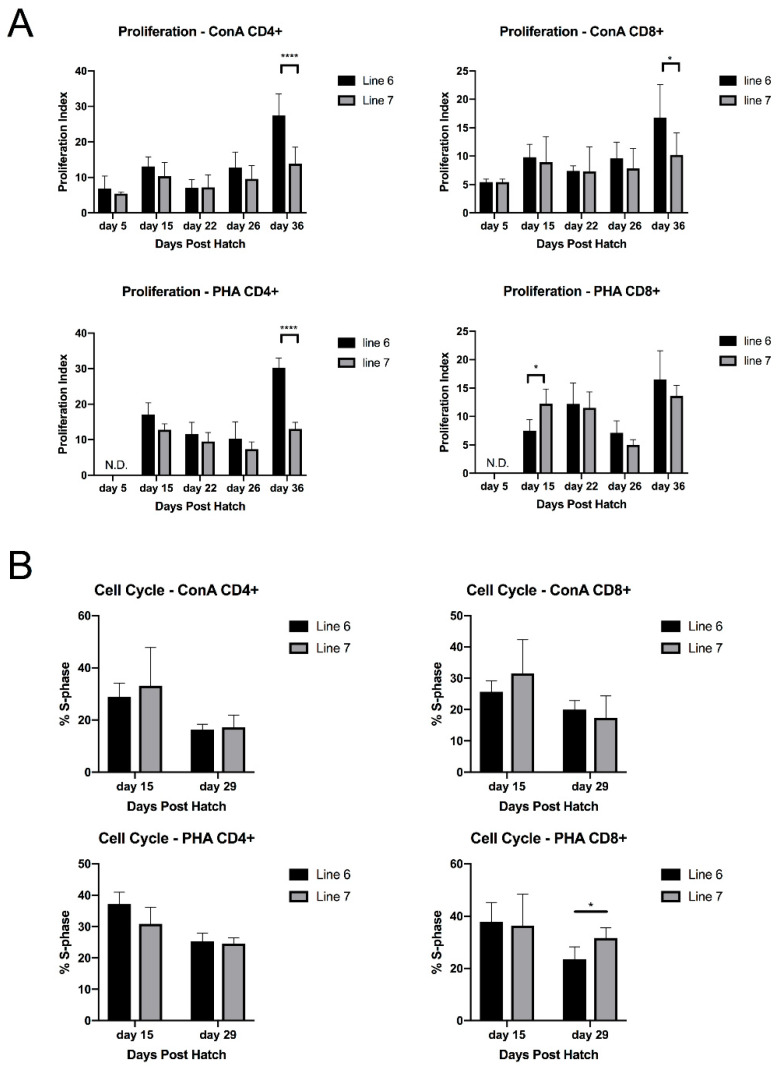
Mitogen responses in MHC-matched lines. (**A**) Early splenic T cell proliferation did not consistently differ between lines 6 and 7 in response to lectin mitogens until 36 days of age. Pooled or individual (depending on age; see methods) white cell fractions from the spleens of chicks at the ages indicated were stimulated with 10 μg/mL of ConA or 100 μg/mL of PHA for 72 h. Sample sizes and pooling within samples at each timepoint are listed in Supplementary Table S1. Groups were compared within assay at each timepoint by Bonferonni-corrected Student’s *t*-test. * *p* < 0.05; **** *p* < 0.0001. N.D. = not done. (**B**) Splenic T cell S-phase fraction did not generally differ between lines 6 and 7 in response to lectin mitogens ConA or PHA, with the exception of PHA-stimulated CD8+ cells at 29 dph. Pooled or individual white cell fractions from the spleens of chicks at the ages indicated were stimulated with 10 μg/mL of ConA or 100 μg/mL of PHA for 48 h. Spleens from 15-day-old chicks were pooled in groups of two; spleens from 29-day-old chicks were assayed individually. *n* = 6 sample pools or individuals per group at each timepoint. Groups were compared within assay at each timepoint by Bonferonni-corrected Student’s *t*-test. Representative of two assays at each timepoint. * *p* < 0.05.

We compared the proliferative responses to lectin mitogens within the splenic CD4+ and CD8+ T cell subsets in uninfected MD-resistant (line 6) and MD-susceptible (line 7) chickens. We separately analyzed the response of CD4+ and CD8+ populations to ConA and PHA stimulation using CellTrace cell labeling for proliferation (Supplementary Figure S1), and confirmed that the proliferation indices of both cell types, as well as the samples in bulk, did not generally differ significantly between lines prior to 36 days of age, except for CD8+ cells in response to PHA in line 7 at 15 days of age; there was, though, a trend toward increased responsiveness in the resistant line. In contrast, CD4+ cells in the resistant line showed a significantly higher response to both mitogens at 36 days of age (Figure 1A). In addition, we performed EdU-incorporation assays for cell cycle analysis and found that the percent of T cells entering S phase after 48 h of stimulation were similar for both lines, except for a mild increase in CD8+ T cells proliferating in line 7 in response to PHA at 29 days of age (Figure 1B; data representative of 2 replicates).

### 3.2. MHC-Matched B2 Haplotype MD-Resistant and MD-Susceptible Lines Differ in TCR2+ and TCR3+ Splenocyte Frequency

**Figure 2:**
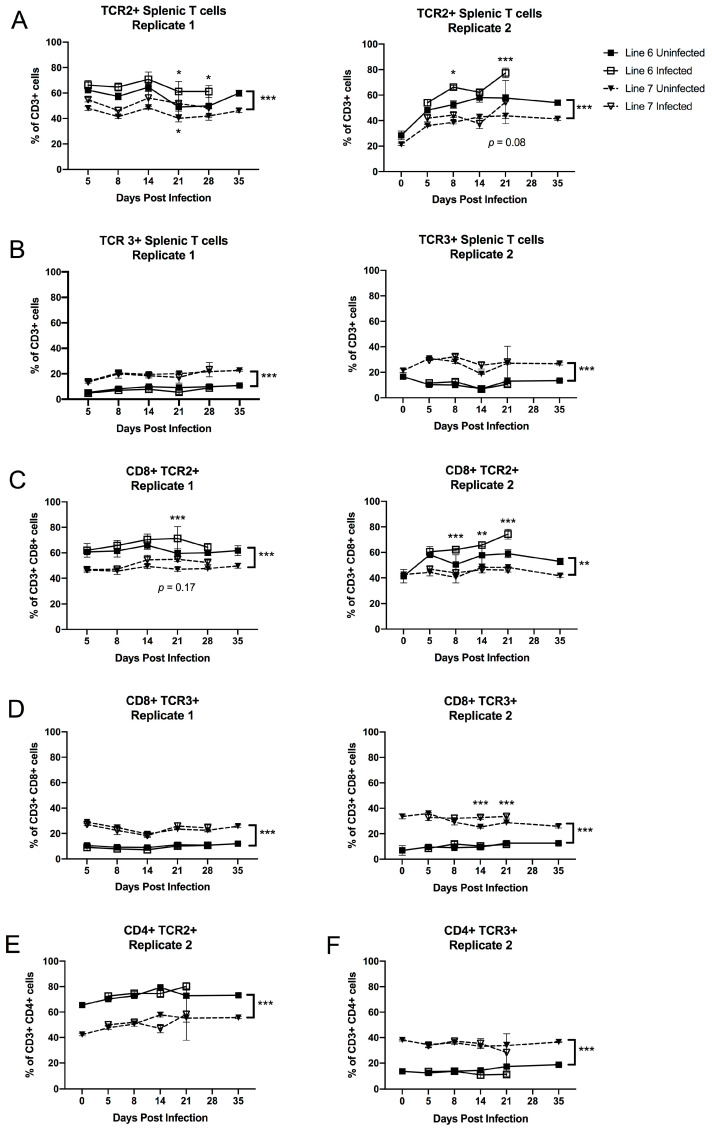
TCR usage in MHC-matched lines. Splenocytes from line 6 and 7 were analyzed at indicated times post-infection with MDV. Resistant birds used TCR2 at a higher frequency, and TCR3 at a lower frequency, within the splenic T cell population than susceptible birds. MDV infection resulted in increases in TCR2 usage in splenic CD8+ T cells of resistant line 6 birds. (**A**) TCR2 usage in splenic T cells; (**B**) TCR3 usage in splenic T cells; (**C**) TCR2 usage in splenic CD8+ T cells; (**D**) TCR3 usage in splenic CD8+ T cells; (**E**) TCR2 usage in splenic CD4+ T cells; and (**F**) TCR3 usage in splenic CD4+ T cells. * *p* < 0.05, ** *p* < 0.01, *** *p* < 0.001. Infected and uninfected birds of each line are compared by Tukey’s test. Line differences by ANOVA are indicated on the right. For replicate 1, *n* = 4 sample pools of 3 birds each per group at day 5; *n* = 4 individual birds per group at days 8, 14, 21 and 28; and *n* = 6 remaining birds per unchallenged control group at day 35. For replicate 2, *n* = 3 sample pools of 3 birds per unchallenged control group at day 0; *n* = 3 sample pools per group at day 5, excepting line 7 uninfected which had 2 pools of 3 birds; *n* = 5 individual birds per group from days 8, 14, and 21; and *n* = 5 and 8 remaining birds per unchallenged control group for lines 6 and 7, respectively, at day 35.

We examined the αβ (conventional) TCR repertoire of splenic cells in line 6 and line 7 chickens with and without MDV infection over time, via flow cytometry (Supplementary Figures S2 and S3). As shown in Figure 2A,B, CD3+ T cells from both lines expressed TCR2 (chicken T cell antigen corresponding to TCRαβVβ1) more frequently than TCR3 (corresponding to TCRαβVβ2), (consistent with previous literature on TCR usage in the chicken [33]); however, line 6 splenocytes were more strongly biased towards TCR2, with 50–65% of their cells expressing TCR2, and only about 10% expressing TCR3. In contrast, 20–30% of line 7 splenocytes expressed TCR3, with a proportional reduction in TCR2 compared to line 6. During infection, the proportion of TCR2+-expressing CD3+ splenocytes increased in line 6 only in replicate 2 (*p* = 0.026 at 8 days post infection), and in both lines in replicate 1 (*p* < 0.05 for both lines by 21 days post infection) (Figure 2A); however, expression of TCR3 was minimally affected by infection status, suggesting that TCR3+ cells may respond poorly to MDV antigens (Figure 2B). In replicate 2, splenocytes were examined on day 0 of infectious challenge, i.e., 1 day of age; interestingly, TCR2+ cells were much less frequent in both lines at 1 day of age, while TCR3+ cell frequency was intermediate between lines at this earliest timepoint and diverged within the first week of life (Figure 2A,B).

We additionally examined the TCR repertoire of CD8+ CD3+ T cells (cytotoxic T cells). In replicate 1, we included antibodies against both the CD8α and CD8β chains in our flow cytometry panel, which allowed us to examine the conventional CD8αβ T cell population, while in the second replicate, we only used the CD8α antibody to identify CD8+ T cells, as CD8αα cells made up only 5–10% percent of the CD3+ population in replicate 1 (we replaced the CD8β marker with CD4 in replicate 2). The between-line differences in TCR usage on CD8+ T cells (*p* < 0.001) were similar to those in bulk T cells. However, upon infection, we observed a significant bias toward higher TCR2+ frequency in the CD8+ T cells of line 6 birds in replicate 2, beginning on day 8 (*p* < 0.05); and in replicate 1, this bias was observed across both lines (*p* < 0.001), but only day 21 in line 6 was individually statistically significant (*p* = 0.002) (Figure 2C). In contrast, TCR3 usage in CD8+ cells was minimally affected in line 6 in both replicates, but was mildly increased in line 7 birds relative to controls after 2 weeks of age only in replicate 2 (*p* = 0.025) (Figure 2D). We examined the TCR repertoire of CD4+ CD3+ T cells in replicate 2. In contrast to the CD8+ T cells, the CD4+ population showed no effect of MDV infection on TCR usage until day 14, at which time non-statistically significant reductions occurred in both TCR2 and TCR3 subsets. By 21 days post-infection, individual birds variably became highly biased toward one or the other subset, as is expected from the development of clonal CD4+ T cell tumors. The lack of a TCR2 or TCR3 response in the CD4+ subset prior to day 21 suggests that the CD4 T cell response was not strongly biased to one or the other TCRβ family (Figure 2E).

**Figure 3:**
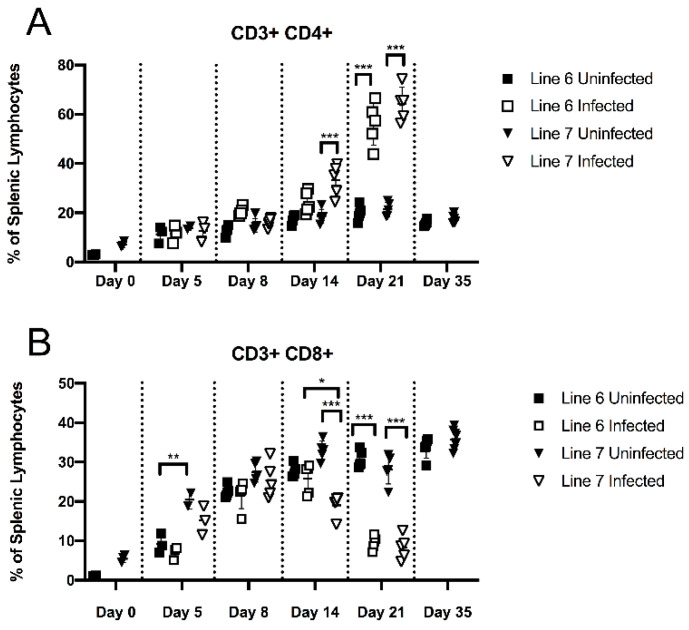
CD4+ and CD8+ T cell percentages in spleen of MD-resistant and MD-susceptible birds. Splenocytes from line 6 and 7 were analyzed at indicated times post-infection with MDV in replicate 2. CD4+ T cells increased and CD8+ T cells decreased in spleens of both lines by 21 days post-infection. (**A**) Percentage of CD3+ CD4+ T cells in total gated splenic lymphocytes. (**B**) Percentage of CD3+ CD8+ T cells in total gated splenic lymphocytes. Infected and uninfected birds within and across lines are compared by Tukey’s test; sample numbers are the same as Figure 2, replicate 2. * *p* < 0.05, ** *p* < 0.01, *** *p* < 0.001.

We compared CD4+ CD3+ and CD8+ CD3+ T cell frequencies within the spleen of line 6 and line 7 birds in replicate 2 (Figure 3A,B). CD4+ and CD8+ T cell frequencies increased in both lines over the first 8 days after hatching, with no effect of infection on these populations until day 14, when CD4+ T cells began to increase; this was significant (*p* < 0.001) in the MD-susceptible line 7 birds. CD4+ T cells were strongly increased in both lines by day 21 with infection (*p* < 0.001), from 16–25% of the splenic lymphocyte population in controls to 44–74% in infected birds (Figure 3A). Conversely, CD8+ T cells were proportionally reduced from 22–32% of splenic lymphocytes in uninfected birds to 5–12% in infected birds by day 21 (*p* < 0.001), with no statistically significant difference between lines in either CD4+ or CD8+ T cell percentage at 21 days post-infection (Figure 3B).

We attempted to assay TCR1 (TCRγδ) in experiment 1, replicate 1; however, our fluorescent secondary streptavidin conjugate gave no specific staining in any samples from days 5–14, including single-color controls, so we removed this marker from later flow cytometry timepoints. We confirmed in single-stain analysis of cecal tonsil lymphocytes from each line and a pooled aliquot of spleen cells from both lines, at day 21, using a different fluorescent conjugate, that the TCR1 antibody itself does bind TCR1+ cells from both lines.

### 3.3. MHC Class II, but Not MHC Class I, Is Differentially Expressed on Splenic Antigen-Presenting Cells from MHC Matched B2 Haplotype MD-Resistant and MD-Susceptible Chickens

**Figure 4:**
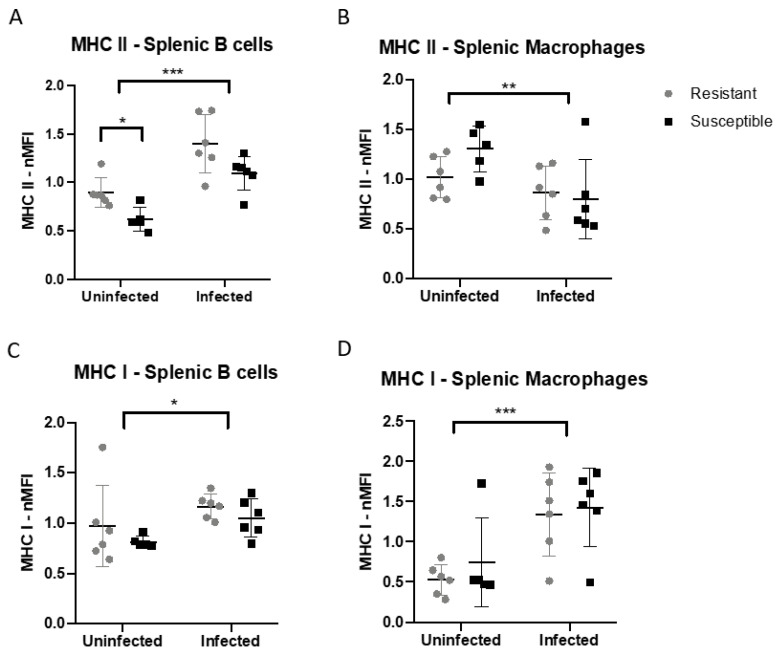
Splenocytes from lines 6 and 7 were analyzed at 7 days of age (7 days post-infection with MDV). MHC class I expression did not differ between lines on B cells and macrophages, but responded to infection; MHC class II differed on B cells and was responsive to infection in both lines. (**A**) MHC class I expression on splenic B cells. (**B**) MHC class I expression on splenic macrophages. (**C**) MHC class II expression on splenic B cells. (**D**) MHC class II expression on splenic macrophages. nMFI: normalized median fluorescent intensity (see methods). * *p* < 0.05, ** *p* < 0.01, *** *p* < 0.001; response to infection is indicated by ANOVA, and differences between lines by post-hoc Tukey’s test. *n* = 6 individual birds in infected groups and uninfected line 6, and 5 in uninfected line 7.

To test the hypothesis that differences in MHC expression on antigen-presenting cells may account for the differences in baseline TCR usage in line 6 and line 7, despite the shared MHC haplotype of these lines, we compared MHC class I and MHC class II expression on splenic B cells and macrophages in at 7 days of age by flow cytometry (Supplementary Figure S4); additionally, we examined MHC expression in MDV-infected chicks at the same age (7 days post-infection), although a lower challenge dose was used in this experiment (500 pfu rather than 2000 pfu; both dosages of vvMDV strain Md5 routinely elicit MD in susceptible birds). We found statistically significant (*p* < 0.05) differences in MHC class II expression, but not MHC class I expression, on splenic B cells in uninfected birds, with the resistant line 6 birds expressing higher MHC class II; with infection, both lines upregulated MHC class II to a similar degree (*p* < 0.001), although the difference between lines was not significant in infected birds (Figure 4A). On splenic macrophages, MHC class II was downregulated in both lines at 7 days post-infection (*p* < 0.01) (Figure 4B). Conversely, MHC class I expression was not statistically different between lines on either cell type, although a non-statistically significant difference in expression on B cells (lower in the susceptible line) was noted with infection. MHC class I was upregulated by infection in both lines on B cells (*p* < 0.01) and macrophages (*p* < 0.001) at 7 days.

### 3.4. TCR Spectratype Analysis Demonstrates early Clonal Responses to MDV Infection in Both MD-Resistant and MD-Susceptible MHC Matched (B2 Haplotype) Birds

**Figure 5:**
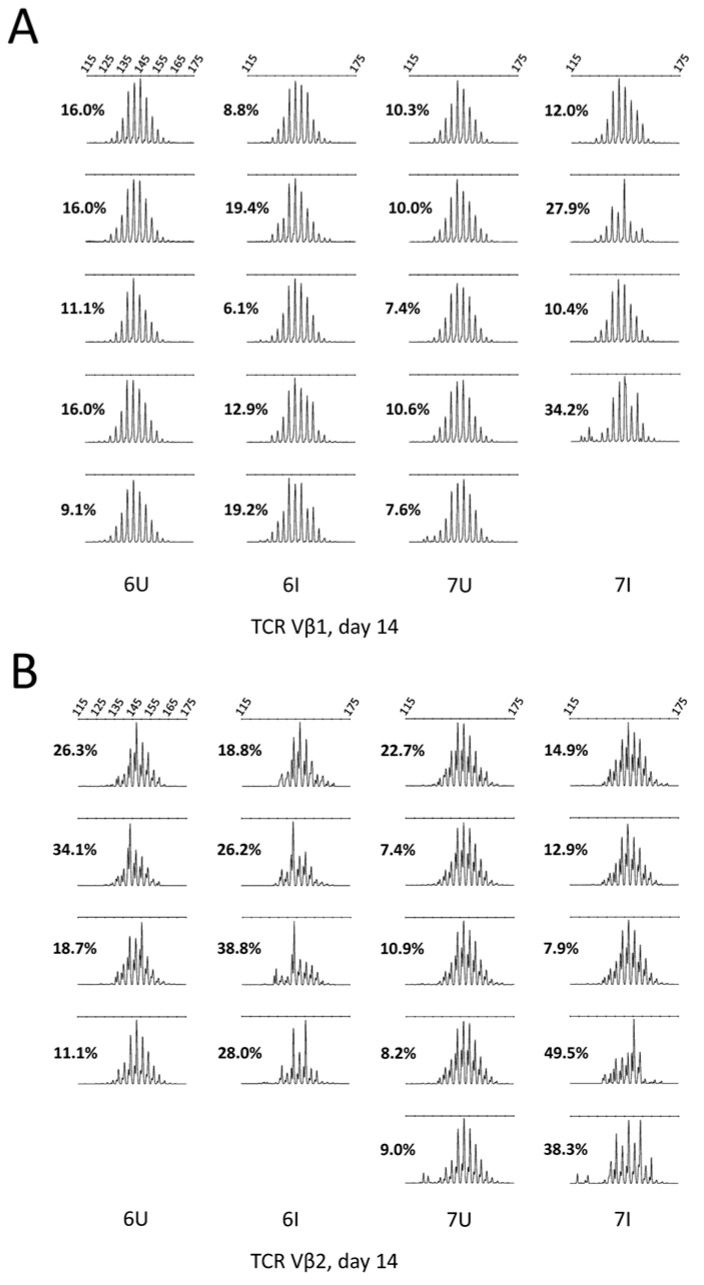
TCR spectratyping of splenocytes from MHC-matched lines. Divergence scores in comparison to controls within each line are indicated as percentages, and horizontal scale axes indicate fragment size. Individual control birds are compared to the remaining controls of the same line. (**A**) TCR Vβ1 and (**B**) Vβ2 PCR fragments at 14 days post-infection with MDV. Each spectratype image represents an individual bird from line 6 or line 7, without MDV infection (columns 6U, 7U) or with MDV infection (columns 6I, 7I); 4 or 5 birds were spectratyped in each group.

We performed TCR spectratyping on splenic tissue from MD-resistant line 6 and MD-susceptible line 7 birds at multiple stages of MDV infection. We were able to identify oligoclonality in bulk TCR Vβ1 and TCR Vβ2 T cell populations in infected birds as early as 14 days post-infection (Figure 5A,B), which can be identified as an increase in total divergence score compared to the control samples, especially in the spleens of susceptible line 7 birds, but also particularly in the TCR Vβ2 subset in resistant line 6 birds. Both lines showed oligoclonality in TCR Vβ1 populations by day 21, but only line 7 showed the development of strong individual clones, consistent with tumor formation occurring only in these birds (Supplementary Figure S5).

### 3.5. MHC Congenic Lines 15I_5_.B21 and 15I_5_.B19 Differ in Use TCR2+ and TCR3+ Frequency in the CD8+, but Not CD4+ Splenocyte and Peripheral Blood Mononuclear Cell Populations

**Figure 6:**
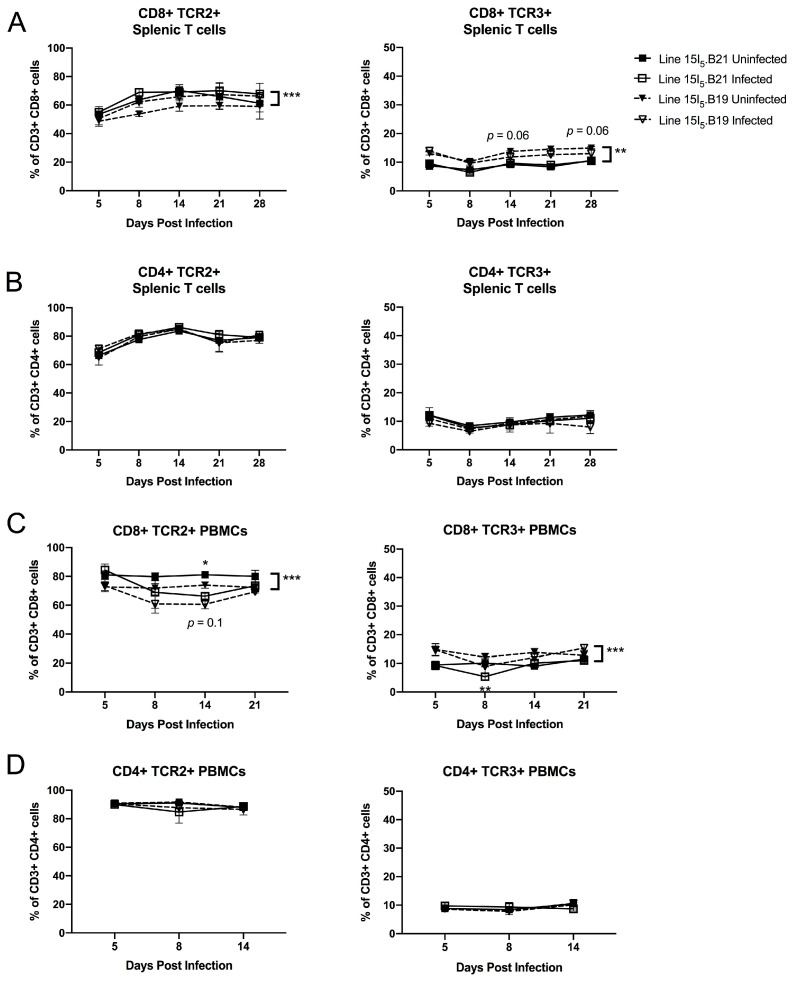
TCR frequency in MHC congenic lines. Days post-infection with MDV are indicated. TCR families were differentially responsive to MHC haplotype and MDV infection in CD8+, but not CD4+, T cells in both splenic and peripheral blood pools. (**A**) TCR2+ and (**B**) TCR3+ frequency in splenic T cells. (**C**) TCR2+ and (**D**) TCR3+ frequency in peripheral blood T cells. * *p* < 0.05, ** *p* < 0.01, *** *p* < 0.001. Infected and uninfected birds of each line are compared by Tukey’s test. Line differences by ANOVA are indicated on the right. *n* = 4 individual birds per group at each timepoint.

Our initial model of genetic host resistance to MD involved inbred bird lines that share the same MHC haplotype (B2) but differ at non-MHC loci. We also compared TCR usage in a genetic resistance model comparing two congenic lines that differ at the MHC locus but share the same genetic background (presumably including TCR loci), in order to demonstrate whether MHC-TCR interactions might play a role in determining genetic differences in T cell immunity (Figure 6A–D). As shown in Figure 5B, we found that CD4+ T cells did not differ in TCR2 and TCR3 frequency between congenic lines 15I_5_.B21 (genetically resistant to MD) and 15I_5_.B19 (genetically susceptible to MD), or in response to MDV infection. As in the line 6 and line 7 birds, TCR2+ T cells make up a much greater percentage of splenic T cell populations (approximately 90% of CD4+ T cells) than TCR3+ T cells (approximately 10% of CD4+ T cells). In contrast, TCR2+ and TCR3+ frequency in splenic CD8+ T cells differed mildly between lines, suggesting that intra-thymic MHC class I (but potentially not MHC class II) has a direct effect on establishing different TCR repertoires in this model of MD resistance (Figure 6A). As in the MHC-matched resistance model, the MD-resistant line tended to use TCR2 at a higher frequency than the MD-susceptible line on CD8+ T cells. Within the CD8+ population, TCR3 showed no significant response to MDV infection, as in the line 6 and line 7 model. However, the TCR2 population was responsive to MDV infection (*p* < 0.001), with line 15I_5_.B19 (susceptible) gradually increasing in TCR2 frequency in CD8+ T cells until day 21 post infection, at which time both lines were essentially expressing this receptor at the same frequency (Figure 6A). We also analyzed the TCR usage on PBMCs in this model, and found that infection resulted in an approximately 10–15% decrease in TCR2 frequency in blood CD8+ T cells within both lines during days 8–14 of infection (*p* < 0.05) (Figure 6C), which may indicate tissue demand for this T cell population, while TCR frequency was not perturbed in CD4+ PBMCs of either line during this phase of infection (Figure 6D).

### 3.6. TCR Frequency in 6C.7 Recombinant Congenic Lines Is Similar to Parental Lines, and TCR2+ Lymphocyte Fraction Correlates with MD Resistance

**Figure 7:**
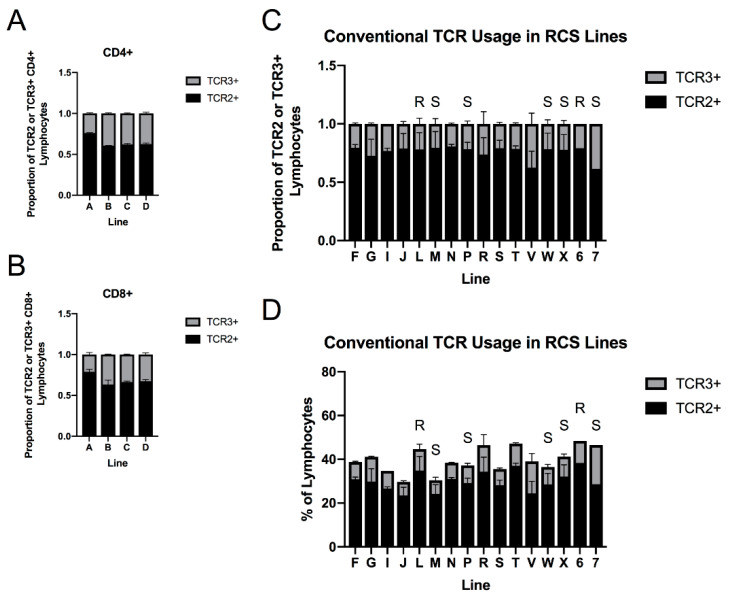
TCR frequency in RCS lines. TCR frequency in RCS lines A-D was compared within (**A**) CD4+ and (**B**) CD8+ peripheral blood mononuclear cell populations. (**C**) TCR frequency within RCS lines F-X was compared within all cells expressing TCR2 or TCR3 within the lymphocyte gate on FSC vs. SSC. Parental strains 6 and 7 are included as controls. Relatively resistant and susceptible lines are indicated with “R” or “S” where known. (**D**) TCR frequency within RCS lines F-X was compared within the total peripheral blood lymphocyte population as gated on FSC vs. SSC. Relatively resistant and susceptible lines are indicated with “R” or “S” where known. Two hens from each line were assayed.

We examined baseline TCR usage within the PBMCs of a panel of line 6 × line 7 recombinant congenic lines (RCS), which have been developed to allow linkage analysis of parental line phenotypes and have been characterized for lymphoid organ size [73], and in the case of several lines, for MD resistance [10,74]. We found that TCR frequency showed little variation, especially in the CD4+ population, and generally proved similar to one or the other of the parental lines (Figure 7A–C). TCR frequency did not correlate with lymphoid organ size, which is known to vary strongly across these lines [73] but does not correlate directly with MD resistance. While TCR frequency within the T cell population did not directly correlate with resistance in the lines with known relative resistance to MD, the fraction of TCR2+ T cells in the total PBMC population was highest in Line 6 and in one congenic line known to be relatively resistant to MD (Figure 7D), suggesting that the role of TCR usage in MD resistance may involve interactions with other factors to determine the available immune repertoire.

### 3.7. MHC-Matched B2 Haplotype MD-ResistantLine 6 and MD-Susceptible Line 7 Encode Differing TCR Variable β1 Genes

We compared the genomic TCR Vβ1 sequences from Illumina DNA-seq data previously generated from pooled blood collected from uninfected MD-resistant line 6 and MD-susceptible line 7 animals. While short (92-bp) read Illumina sequencing does not provide sufficient read-length to uniquely map reads to Vβ genes, we estimated diversity at the Vβ1 locus by aligning all Vβ1-mapping reads to a single model Vβ1 gene and calling variants (Supplementary Data File S1). Interestingly, 62 variants in Vβ1 could be identified in line 6, in contrast to 46 variants in line 7, using a short, 3-bp haplotype window in Freebayes. When more complex haplotypes were considered (10-bp haplotype window), a large proportion of variants (29% of 41 variants) in line 6 were found to have more than three non-reference alleles (Supplementary Data File S1), while only 15% of 34 variants in line 7 had this many non-reference alleles, indicating that there is greater variation present in the TCR Vβ1 sequences of line 6.

A variant hotspot located at the hypervariable CDR1 loop allowed manual identification of longer (85-bp; 27-amino acid) haplotypes in spanning reads; interestingly, seven unique Vβ1 haplotypes could be identified in line 6, while only four unique haplotypes could be identified in line 7 at this site, with one additional haplotype shared between lines. We modelled these CDR1 sequences in the context of a published TCRβ chain (GenBank ABU93634.1), using a crystallography-supported mouse TCR-pMHC complex as a modelling framework; chicken TCRβ adopted the expected two-immunoglobulin domain structure (Supplementary Figure S6). CDR1 sequence affected the predicted shape of the CDR1 loop and its interaction with the CDR3 loop (Supplementary Figure S6), and the aromatic amino acid Trp44 was substituted for Arg44 in the CDR1 loop of five out of eight Vβ1 haplotypes in line 6, versus two out of five haplotypes in line 7 (residue 44 is shown in fuchsia in Supplementary Figure S6).

**Figure 8:**
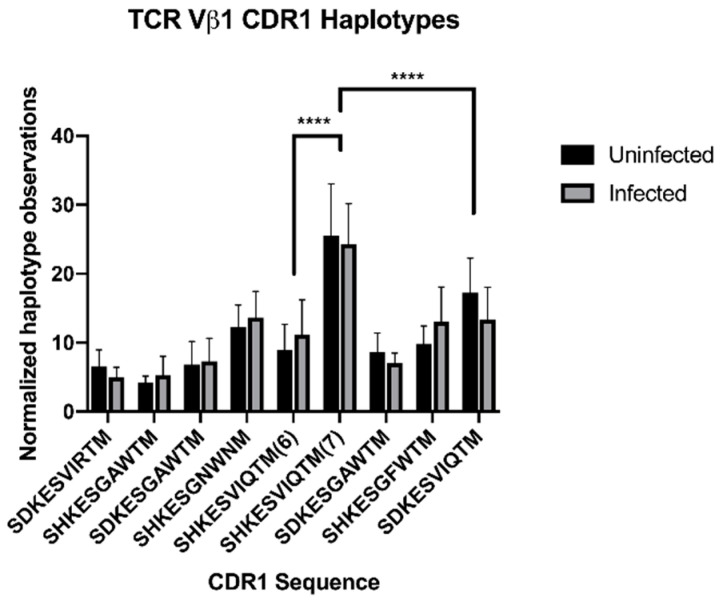
TCR Vβ1 CDR1 haplotypes in line 6 × 7 F_1_ RNA-seq data. Illumina RNA-seq was performed on splenocytes from uninfected birds or birds infected at dpi with MDV. CDR1 haplotypes were predicted with Freebayes. Parental line 6 and 7 alleles contributing the SHKESVIQTM haplotype could be differentiated by a downstream Val-Leu-53 substitution in the C-C′ loop. No significant difference was observed between uninfected and infected samples, so haplotypes were compared inclusive of infection status by Tukey’s test. **** *p* < 0.0001. *n* = 7 infected and 7 uninfected birds.

In order to estimate TCR Vβ1 gene usage at the mRNA level, we compared the usage of Vβ1 CDR1 haplotypes in a pre-existing Illumina RNA-seq dataset from MDV-infected and uninfected spleens of first-generation (F_1_) hybrids of line 6 and line 7, at 4 days post-infection (18 days of age) [28]. Using Freebayes, we identified nine different 32-bp (ten-amino acid) haplotypes within the CDR1 site, each uniquely identifiable as one of the twelve haplotypes identified by DNA sequencing (Supplementary Figure S6), and estimated usage of each haplotype within the sample (Figure 8). At 4 days post-infection, there was no significant change in usage of any TCR Vβ1 gene within the total TCR Vβ1 pool between infected and uninfected birds. However, the CDR1 amino acid sequence “SHKESVIQTM” (with glutamine falling at position 44) was over-represented in both infected and uninfected samples, with a haplotype from the line 7 parentage encoding this sequence comprising approximately 25% of haplotype observations. Surprisingly, a line 6 haplotype that also encoded this sequence (with a synonymous SNP) was observed less than half as frequently; the only definitive difference between these two haplotypes that could be inferred from the longer DNA-based haplotype is a Val-Leu substitution at position 53 in the variable region’s C-C’ loop (which is expected to form part of the Vα-Vβ interface), although it is possible that these TCR Vβ1 genes contain important sequence differences further from the CDR1 region, e.g., at the CDR2 or CDR3 loops. Three CDR1 haplotypes identified in the DNA sequence data (two from line 6 and one from line 7) were not observed within the RNA sequence data, at the detection threshold used in our analysis.

Additionally, one of the line 6 TCR Vβ1 genes contained a four-base deletion that introduces a Gln to Ile (polar to hydrophobic) amino acid change at position 111, just upstream to the V-D junction of the CDR3 loop (Supplementary Figure S7). Using the RNA-seq dataset from splenocytes of 4-day-old line 6 × 7 F_1_ hybrid birds either uninfected or infected with MDV at hatch, we observed approximately 69% usage of the canonical Gln-111 if the upstream codon was unmodified, while Ile-111 was used 4% of the time. In this sequence context, usage of Gln-111 was reduced during early MDV infection, while Ile-111 usage remained stable, as did usage of a codon-initial adenine at this position, which was present in about 11% of sequences (Supplementary Figure S8).

**Figure 9:**
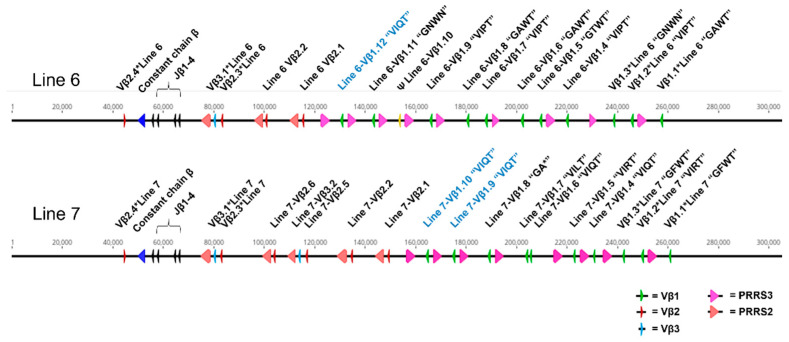
*TCRβ* loci are divergent in line 6 and line 7 chickens. PacBio DNA-seq de novo assemblies were built for MD-resistant line 6 and susceptible line 7 and used to map the *TCRβ* loci. Number of probable gene segments and location with the locus is shown; variable segments are annotated as alleles (e.g., “Vβ1.1*Line 6”) only where in-locus arrangement is well-preserved between lines. Trypsinogen (PRRS2 and PRRS3) genes were found associated with the variable segments. Abbreviated CDR1 region haplotypes are indicated for the Vβ1 sequences. “GA*” indicates a CDR1 motif with an apparent single base deletion and an in-frame stop codon, which may be due to sequencing error. One longer CDR1 haplotype (light blue; “SHKESVIQTMF”) was shared between lines. Ψ indicates pseudogene.

Finally, we were able to obtain long-read (~20 kb at N50) PacBio DNA sequencing data from both lines 6 and 7. De novo assembly in Canu allowed reconstruction of the *TCRβ* locus for each line (Supplementary Figure S9A,B). Despite the repetitive nature of the locus, line 7 could be constructed successfully (with all *TCRβ* genes in one contig) using stringent defaults for PacBio data (4.5% between-read allowable error rate), and contained ten Vβ1 genes and six Vβ2 genes (Figure 9, Supplementary Figure S9B). In contrast, line 6 required somewhat less stringent parameters to construct a single *TCRβ*-containing contig (10.5% allowed error rate), and contained at least twelve Vβ1 genes and only four Vβ2 genes (Figure 9, Supplementary Figure S9A), consistent with the differential TCRβ usage identified by flow cytometry. One line 6 Vβ1 gene appeared to contain a large indel (14 novel bases replacing 171 bp of sequence), and is therefore most likely a pseudogene. A recently identified putative Vβ3 gene was present in both lines; one copy was present in line 6, similar to what was predicted for the Red jungle fowl reference [45] while line 7 contained a second copy in apparent segmental duplication with the nearest Vβ2 gene (Figure 9). 

Similarly to the Illumina data, CDR1 haplotypes were identified for each Vβ1 gene in both lines (shown in Supplementary Figure S10); most previously identified haplotypes could be identified within the assembled PacBio data, with the exception of SHKESVIRTMF and SHKESGFWTMF in line 6. Conversely, genes containing the haplotypes SHKESGTWTMF in line 6 and SDKESVILTMF in line 7 were present in the assembled PacBio data but were not previously identified in the Illumina data; these may contain sequencing errors and their sequences should be confirmed by targeted resequencing. The most common CDR1 haplotypes in line 7 were SDKESVIRTMF, SDKESVIQTMF, and SHKESVIQTMF, each of which was represented by two genes. In line 6, SHKESVIPTMF was represented by three genes, and SDKESGAWTMF was represented by two; the “VIQT” motif was only represented by one gene in line 6. Figure 8 shows the reconstructed model of the *TCRβ* loci in line 6 and 7, with the single shared CDR1 haplotype in Vβ1, SHKESVIQTMF, highlighted between the lines; coding sequences for Vβ1 and Vβ2, and full sequences for putative Vβ3, are provided in Supplementary Figures S10–S12.

### 3.8. MDV Downregulates TCR Surface Expression Differentially in MHC-Matched B2 Haplotype MD-Resistant and MD-Susceptible Lines

**Figure 10:**
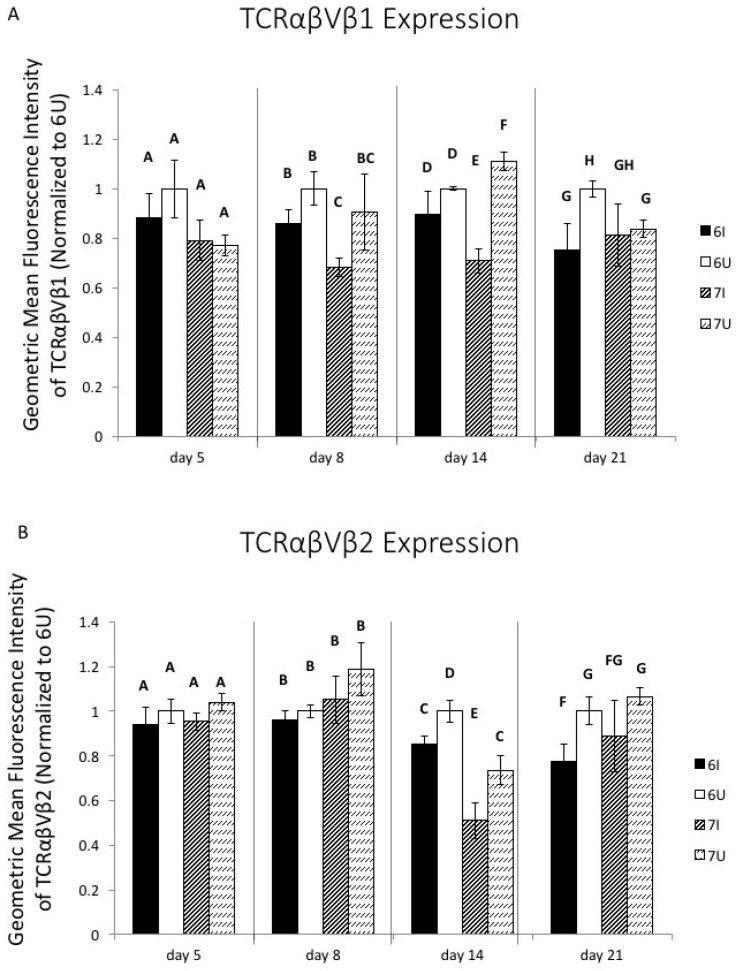
TCRαβVβ expression on CD3+ splenocytes. Geometric mean fluorescence intensity of (**A**) TCRαβVβ1 (TCR2) and (**B**) TCRαβVβ2 (TCR3) staining of gated CD3+ TCR+ cells from samples shown in Figure 2A,B, replicate 1. Non-overlapping letters indicate significant difference within days by *p* < 0.05 on Bonferonni-corrected Student’s *t* test.

Levels of TCR surface expression on gated bulk TCR+ CD3+ T cells were examined by flow cytometry in experiment 1, replicate 1. In both lines, a mild reduction of TCRαβVβ1 surface expression by TCR2 antibody labeling was seen on day 8; however, this became statistically significant in line 7 on day 14, while it was not significant in line 6 until day 21 (Figure 10A). TCRαβVβ2 surface expression by TCR3 antibody labeling was significantly reduced in both lines by day 14 (Figure 10B).

### 3.9. TCR Expression Is Reduced in an MDV-Reactivated Tumor Cell Line

**Figure 11:**
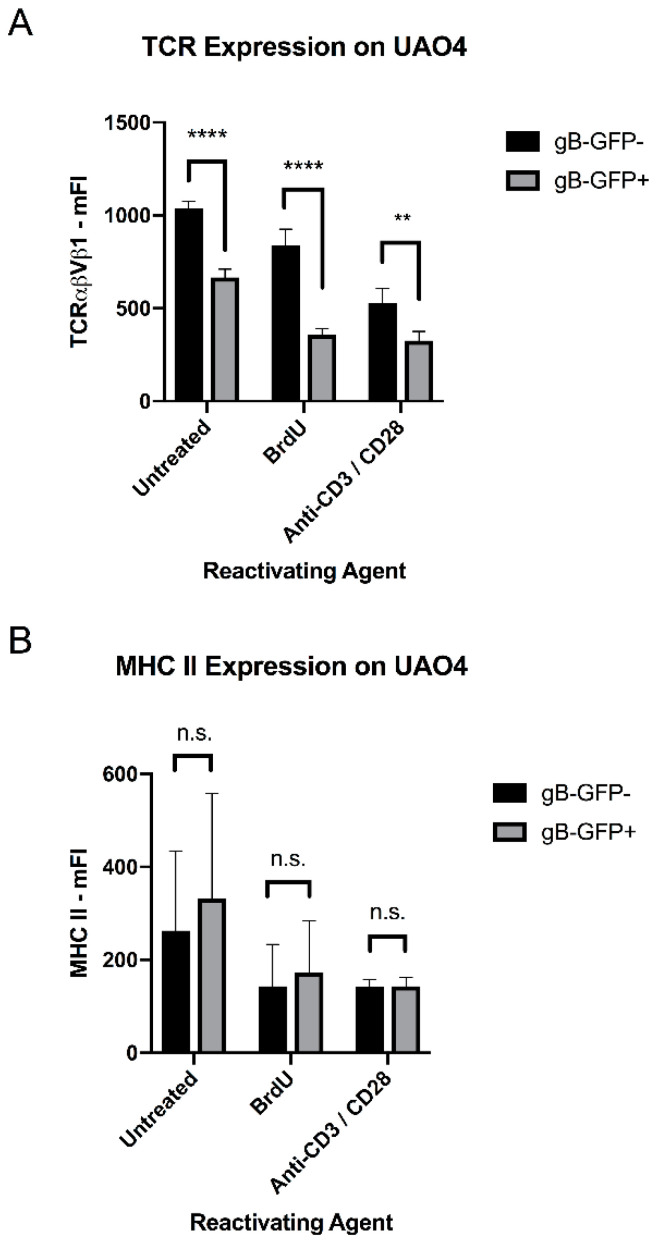
Reactivating MDV downregulates TCRαβVβ1. (**A**) Reactivating gB-GFP+ UAO4 cells downregulates surface expression of TCRαβVβ1, measured by TCR2 antibody fluorescence in flow cytometry. gB-GFP+ and gB-GFP- cells were gated within untreated, BrdU-treated, and anti-CD3/CD28 antibody-treated samples (averaged across three antibody dilutions, as reactivation was not concentration-dependent within the range tested). (**B**) Reactivating gB-GFP+ UAO4 cells express similar levels of MHC class II to latently-infected gB-GFP- UAO4 cells within the same sample, as measured by flow cytometry. *n* = 3 parallel-treated cell culture wells per treatment. ** *p* < 0.01, **** *p* < 0.0001 by Sidak’s multiple comparisons test. n.s. = no significant difference. mFI = mean fluorescence intensity.

We examined the levels of TCRαβVβ1 expression on the TCR2+ CD4+ T cell tumor line UAO4, which contains latent, reactivatable MDV and expresses gB-GFP upon MDV reactivation with bromodeoxyuridine (BrdU; a thymidine analogue), soluble anti-CD3 and anti-CD28 antibodies (e.g., through TCR-dependent signaling) (Figure 11A), phorbol myristate acetate (PMA; a potent activator of protein kinase-C), or any of several interleukin cytokines (personal communication, Henry Hunt, USDA-ADOL, retired), but not anti-TCR2 antibody alone (Supplementary Figure S13A,B). We found that the small number of reactivating UAO4 cells present in the absence of any treatment also express lower levels of TCRαβVβ1 (although our staining protocol for flow cytometry increased reactivation and thus can be considered a treatment). Either BrdU or the combination of soluble anti-CD3/CD28 antibodies reduced TCRαβVβ1 expression on UAO4 cells, with the GFP-expressing cells showing lower TCRαβVβ1 expression than GFP-negative cells (Figure 10A). In contrast, MHC class II was not significantly affected by either treatment or in GFP-expressing cells relative to GFP-negative cells (Figure 10B), and the TCR downregulation was not caspase-dependent, as the caspase inhibitor Z-VAD-FMK did not affect it (Supplementary Figure S13C).

## 4. Discussion

We broadly characterized the T cell repertoires and responses of MD-resistant and MD-susceptible chickens using two pairs of inbred chicken lines, in order to identify immune responses that correlate with a resistant phenotype in vivo. Initially we hypothesized that intrinsic activation capacity in peripheral (i.e., splenic) T cells might differ between lines in our MHC-matched model, in accordance with an early hypothesis that T cell infection relies on activation [75] and, thus, susceptible birds may have more easily activated T cells in general; differences in mitogen response have been historically observed and studied in inbred chicken lines [76,77,78], including lines 6 and 7 [30,31,79]. At early timepoints, CD4+ cells did not significantly differ in proliferative response to mitogen stimulation, although there was a trend toward increased responsiveness in the resistant line; however, by 36 days of age, differences in both CD4+ and CD8+ responses to ConA became highly significant, which may indicate an age-acquired advantage in mitogen response in the resistant line. These data are in contrast to previously reported whole blood assays which indicated a bulk proliferation advantage in the susceptible line, unless differences in cell count were accounted for (in which case line 6 appeared more responsive to PHA only) [30,31]; but in agreement with previously reported purified splenic lymphocyte mitogen responses, which found no consistent advantage in bulk responses to PHA in healthy birds [31,79]. Strong differences in lymphocyte count between lines likely account for the differences between these assays, along with the presence or absence of response-modulating non-lymphocyte cell populations in sample preparations [30,79]. Our data, which extended previous findings about peripheral T cell proliferative capacity to CD4+ and CD8+ subpopulations, suggests that activation ability alone, early in infection in young chicks, may not explain differences in susceptibility between these lines, and that either the numbers of lymphocytes present, interactions with non-lymphocyte cell populations, or the TCR repertoire itself are likely to be responsible for differences in response to MDV infection or development of lymphoid tumors. We note that we see a similar decrease in PHA response around 26 days of age as that reported for line 6 in Fredericksen et al. 1983 [30], which could indicate developmental effects on expression of PHA-binding glycoproteins, or on either numbers or function of regulatory T cells, the latter of which are known to have high lectin affinity in humans and mice [80]; age-related effects on mitogen responses have also been observed in turkey poults [81].

We identified differences in the usage of the two TCR Vβ families in the T cell repertoire of both our MHC-matched and MHC-congenic MD-resistance models. In our MHC-matched model, in which the same MD-susceptible MHC haplotype has been maintained in inbred lines divergent for disease resistance [10,82,83], we infer these differences, which are as high as 25–30%, may be due to structural differences at the *TCRβ* locus itself, and in fact we were able to identify differences in number and sequence of genes at the *TCRβ* locus in these lines. While we cannot rule out a potential influence of differences in MHC II expression on shaping the TCR repertoires of these lines, MHC class I was expressed similarly on antigen-presenting cells of both lines, making it less likely that the baseline TCRβ usage differences seen in both CD4+ and CD8+ T cells of these lines are due to T cell selection. In addition, we cannot rule out an influence of MHC genes located within the non-classical *Rfp-Y* locus on thymic or peripheral T cell selection, as *Rfp-Y* haplotype differs between these lines. *Rfp-Y* is not linked to MD resistance in this model [15], and one or more of the class I-like genes in this locus appears to be involved in presentation of non-peptide (e.g., lipid) antigens [12,84], but their involvement in selection or activation of T cell subsets remains to be studied. In contrast, our MHC-congenic lines, which share the same genetic background (presumably including TCR and *Rfp-Y* loci) but carry MHC haplotypes linked to different levels of MD resistance, showed mild (5–15%) differences in TCR usage in the CD8+ T cell population only, indicating that MHC class I, but not necessarily MHC class II in this model, shapes different T cell repertoires during thymic selection that are recognizable at the bulk (gene family) expression level. This may help explain the critical importance of the MHC locus as the strongest genetic determinant of host resistance against MD, in addition to differences in quantity of MDV antigen presented by differing MHC alleles [14]. Genetic selection for MHC structures that efficiently present viral peptides is believed to be a critical aspect of immune locus evolution, and in the chicken it has been theorized that MHC class I and the TAP peptide transporter have co-evolved to optimize responses to pathogens such as herpesviruses in the context of a greatly reduced MHC locus [39,41,42,43]. It is additionally likely that TCR loci, of which at least the *TCRβ* locus appears reduced in gene number in the chicken relative to mammalian *TCRβ* loci, are also co-evolving along with their MHC binding partners, and selection for pathogen resistance or susceptibility may skew TCR repertoires towards or away from “best fit” receptors that recognize viral peptides as presented on MHC.

We identified significant shifts in TCR2+ and TCR3+ T cell subsets during MDV infection in our flow analyses. In both of our models, we saw little change in TCR usage within CD4+ populations prior to late infection when CD4+ T cell lymphomagenesis was likely to be occurring. However, in our MHC-matched model, TCR2+ responses visible in the splenic lymphoid population as a whole could only be attributed to CD8+ and not CD4+ T cells, indicating that the shift in CD8+ T cells toward TCR2 in the MD-resistant line 6 was large, even though the relative contribution of CD8+ T cells to the splenic T cell pool decreased significantly during the course of infection. Our measurements are based on relative cell population frequencies; therefore we cannot definitely state whether the shifts seen in TCR usage within the CD8+ population are due to expansion or recruitment of TCR2+ CD8+ cells, or loss of another TCR-bearing subset. Prior literature suggests that TCRαβVβ1+, but not TCRαβVβ2+ T cells are important for the development of anti-MDV serotype 2 (non-oncogenic MDV) vaccinal immunity [85], and analysis of T cell clonality in MD tumors has identified vigorous TCR Vβ1 clonal responses within intra-tumor CD8+ subsets [86]. Our results suggest that TCR2+ responses, specifically within the CD8+ T cell subset, may be playing an important role in the host immune response to MD in this genetic resistance model as well. It is important to note, however, that the strength of such a response does not necessarily correlate with protection against disease; in our experiments, where a high challenge dose was used in unvaccinated birds (2000 pfu of very virulent Md5), most birds that were not euthanized for tissue collection succumbed to the acute infection in the third or fourth week after challenge, regardless of genetic background, similarly to previously reported experiments with highly pathogenic MDV in these lines [83], even though the line 6 birds did not develop apparent tumors. It is possible that a robust anti-viral or anti-tumor response in the line 6 birds contributed to mortality due to cytokine storm rather than providing protection from acute disease.

Additionally, we were able to identify clonality within the TCR Vβ1+ and TCR Vβ2+ subsets directly, using TCR spectratyping analysis. While both resistant and susceptible lines showed increased oligoclonality in MDV-infected birds by day 14 post-infection, this occurred primarily in the TCR Vβ2 subset in the resistant line, while the susceptible line showed early oligoclonality in both TCR subsets. The unusually high oligoclonality in TCR Vβ2 in the MD tumor-resistant line 6 is most likely due to the very low usage of TCRαβVβ2 in this line, magnifying the effect of T cell expansion in response to viral antigens or deletion of infected subsets; however, it is possible that we are detecting early expansion of tumor cells within the small TCRαβVβ2+ T cell population in line 6, which can subsequently be controlled by antiviral or anti-tumor immunity. By day 21 post-infection, very high clonality consistent with tumor development was visible in the spleens of susceptible line 7 birds, while resistant birds showed a high deviation from discrete-normality but no strong clones in either TCR subset, more consistent with a robust T cell response. High oligoclonality has been previously observed in the CD8+ T cell subsets in birds with (CD4+) MD tumors, within both spleen and tumor tissue [86], indicating that this is a feature of the cellular immune response to either viral or tumor antigens, but one that does not directly correlate with protection, since it is observed in birds that develop tumors. Notably, in Mwangi et al. 2011 [86], TCR Vβ1 was the most biased subset in responding CD8+ T cells, including a public sequence that was present in the tumors of several birds. In the future, repertoire analysis of sorted CD4+ and CD8+ T cells from MD-resistant birds will give additional indication of whether T cell responses in these birds may be associated with line-specific public clones. Recently, line-specific public clones were identified in several chicken TCR gamma subsets [87] although it is unknown whether these clones have important antigenic targets such as pathogens or tumors; and public TCR Vβ1 clones were also identified in the intestinal CD8+ T cells of SPF chickens infected with *Eimeria* [88]. Such public clones may represent convergently recombined, germline-determined CDR3 sequences that confer early protection against pathogens, especially in very young animals prior to full TCR repertoire diversification [89,90,91].

We hypothesized that the relatively high usage of TCRαβVβ1 in line 6, visible both in the flow cytometry results and as reduction in clonal diversity in TCR Vβ2 in TCR spectratyping, may be due to heritable structural differences in the TCR Vβ locus in this line. Therefore, we attempted to genetically map the TCR usage trait using a panel of RCS strains between our two MHC-matched inbred lines; we were unable to uniquely identify a genomic region across the *TCRβ* locus segregating across these lines in the same pattern as TCR usage within historically collected microsatellite data [10], collected at four full-sibling crosses into inbreeding. However, at the time of microsatellite typing, these lines were not fixed, and several low-producing lines were subsequently rescued with backcrossing to line 6, so additional typing of genetic markers in these lines would be necessary to perform a valid linkage analysis. 

We were able to draw inferences about the heritability of the TCR usage trait (which appeared similar to parental lines as would be expected from a biallelic Mendelian trait) and also to compare the usage patterns with known MD susceptibilities in several of the RCS lines. While most lines followed the TCR usage phenotype present in the resistant line (unsurprisingly, as these RCS lines were developed through back-crossing to line 6), the several lines known to be relatively susceptible to MD showed lower percentages of conventional T cells overall within the peripheral lymphocyte pool, regardless of the distribution of TCR frequency within T cells. This could indicate that not only the TCR locus, but also factors that affect over-all T cell numbers, influence the availability of cells responsive to infection. A rigorous comparison of the immune profiles of these RCS lines in MDV infection has not been completed, to our knowledge, but would help answer questions about the relative contribution of these phenotypes to MD resistance; unfortunately these lines are no longer being maintained (personal communication, R. Kulkarni, USDA-ADOL).

Although we lacked a robust structural model of the *TCRβ* locus for lines 6 and 7, we were able to compare the gene diversity present in the TCR Vβ1 family in our MHC-matched MD-resistant model, using both standard Illumina DNA-seq and long-read PacBio DNA-seq. Our PacBio assemblies of the *TCRβ* locus were overall organized similarly to recently assembled Red jungle fowl assemblies [45,46], containing adjacent Vβ1 and Vβ2 regions with individual variable genes in apparent segmental duplication with PRRS2 and PRRS3 trypsinogen genes, four J chain segments in each line, and a single constant chain followed by a reverse-oriented Vβ2 gene [45]; however these assemblies differed in the number of variable genes in each line, from each other and from either jungle fowl assembly. Notably, the jungle fowl assembly from Liu et al. 2020, which was sequenced from BAC clones [46], contains only four Vβ1 genes; this might indicate loss of sequence within one or more clones in the CHORI-262 BAC library, as both our inbred layer assemblies and the jungle fowl assembly from Zhang et al. 2020 [45] contained substantially more Vβ1 gene segments (11 in the assembly from Zhang et al. 2020; 12 and 10 in our line 6 and 7 assemblies, respectively). In addition, while both jungle fowl assemblies identified four Vβ2 genes, we found six in line 7 and four in line6, further indicating that chicken *TCRβ* haplotypes can contain differing numbers of gene elements within the locus, similarly to structural *TCRβ* locus rearrangements recently inferred in humans [92] and known structural rearrangements of human IGH loci [93,94,95]. Finally, we note the presence of a putative Vβ3 gene, from which rare transcripts were found in rearrangement with Jβ4 in 5′ RACE analysis by Zhang, et al. 2020 [45]; this segment was duplicated along with its flanking Vβ2 and PRRS3 genes in our line 7 assembly.

We noted that in terms of single nucleotide polymorphism (SNP) density, line 6 was more divergent from the Red jungle fowl reference sequence for Vβ1, and also exhibited a larger number of multi-variant haplotypes, indicating more inter-gene diversity and potentially more variable gene blocks present within the Vβ1 family, and fewer within the Vβ2 family which was confirmed by long-read sequence analysis. Additionally, we also identified a four-base deletion in one TCR Vβ1 gene in our MD-resistant line, which is likely to affect affinity and usage of that Vβ1 gene. As this deletion occurred just upstream of the V-D junction in the CDR3 loop of this TCR, it is likely to produce an in-frame product after VDJ recombination; however, unless nucleotides are deleted upstream of the V-D junction, the canonical Gln-111 that is present in all other TCR Vβ1 genes will be substituted with Ile-111. In *TCRβ* RNA sequence data from line 6 × 7 F_1_ splenocytes, the canonical Gln-111 was maintained 69% of the time during codon end processing if the upstream codon was present (and greater than 50% of the time regardless of upstream codon modification). We modelled TCRβ-chains in the context of both the original sequence and the substituted Ile-111 in Swiss-Model (Supplementary Figure S14) and found that this substitution could affect the predicted orientation of the CDR3 loop, which is consistent with comparatively rare usage of Ile-111 in normal spleen *TCRβ* RNA sequence data obtained from 6 × 7 F_1_ birds. Surprisingly, usage of the canonical amino acid was mildly decreased during early infection, indicating either that the canonical TCR-expressing cells make better targets for MDV infection or are less responsive than some other TCR subset(s) at this phase. We speculate that the line 6 repertoire may have been further shaped by inclusion of a non-canonical variant which is not outcompeted by other early responding T cells, or perhaps is less susceptible to lytic infection.

Additionally, we noted nearly twice as many variants present within the CDR1 hypervariable region of Vβ1 in line 6 as line 7, and noted changes in both the structure of haplotypes and their selection in the peripheral repertoire, suggesting that there are functional differences between the CDR1 regions in each line. The CDR1 region is thought to primarily bind to MHC, although it may also interact with bound peptide [96]. Notably, line 6 has an expanded repertoire of CDR1 loops containing a hydrophobic aromatic residue at position 44, which is not present in the most commonly selected variant in line 6 × 7 F_1_ birds. Conversely, the most commonly used CDR1 variant (SHKESVIQTMF) in line 6 × 7 F_1_ birds, which is overrepresented at 25% usage, is a line 7 haplotype; the corresponding line 6 haplotype is not similarly overrepresented and also contains a downstream substitution in the Vα-Vβ interface. While the presence of two genes in line 7 containing that haplotype could explain its over-usage in F_1_ birds, two other duplicated genes in line 7 were not similarly overrepresented. These data suggest that the line 6 TCR Vβ1 repertoire has been selected away from a “best-fit” relationship between the SHKESVIQTMF motif and at least one of the B2-encoded MHC molecules; this could provide increased protection from MD either by maximizing the use of different TCRs that better recognize MD antigens, especially on MHC class I, or by reducing the availability of activated CD4+ target cells for MDV infection.

Lastly, we studied the influence of MDV on TCR expression. MDV has been shown to down-regulate TCR signaling pathways in MD lymphoblastic cell lines using transcriptome sequencing [97]. Several other herpesviruses, including HHV-6 and HVS, have been demonstrated to directly downregulate expression of the TCR complex, through targeting TCR complex proteins to the lysosome [98,99,100]. We demonstrate here that MDV down-regulates TCRβ expression on splenic T cells during the early (cytolytic) phase of infection, to a greater degree in MD-susceptible birds than MD-resistant birds. As we could not differentiate between the several possible mechanisms of TCR down-regulation in our in vivo model (including direct viral effects, activation of T cells, or expansion of TCR-low tumor cells), we examined the levels of TCRα/β expressed on an MD lymphoblastic cell line with and without viral re-activation. In this in vitro model, we found that MDV reactivation from latency leads to a drop in TCR expression, consistent with a viral evasion strategy that involves downregulating the TCR on infected T cells. This downregulation was stronger than the TCR downregulation that occurred in non-reactivating cells treated with anti-CD3/CD28 antibodies (Figure 11A); further work will be necessary to determine what cell pathways are affected by MDV to cause TCR downregulation, and whether differences in the TCR repertoire between lines 6 and 7 play a role in the differential susceptibility to TCR downregulation in vivo.

While the MHC is the only single locus of large effect on MDV resistance found to date, we have examined an MHC-matched resistance model to identify other potentially interacting factors that may have been selected for in the development of highly MD-resistant chicken lines. The TCR repertoire is a complex trait that is shaped by *TCRα*, *TCRβ*, and MHC loci, as well as self and environmental antigens. We have examined the most tractable of these, *TCRβ*, and found indications that selection for resistance and susceptibility to MDV in an MHC-matched model has modified the repertoire of MHC ligands, i.e., the TCR repertoire. Intuitively, rational breeding strategies to take advantage of MHC-linked resistance to MDV could also incorporate TCR repertoire optimization; further work will be required to develop these kinds of strategies for creating highly disease-resistant poultry stock.

## Data Availability

Sequence data reported in this study have been uploaded to the NCBI Sequence Read Database: long-read PacBio DNA sequencing, Bioproject #PRJNA574234; short-read Illumina DNA sequencing, Bioproject #PRJNA574415. Previously reported RNA sequencing data analyzed in this study are available at NCBI SRA Bioproject #PRJNA574432.

## Supplementary Materials

The following supporting information can be downloaded at: https://www.mdpi.com/article/10.3390/v15030607/s1, Figure S1: Proliferation assay gating and modeling; Figure S2: Flow cytometry gating strategy for experiment 1 replicate 1; Figure S3: Flow cytometry gating strategy for experiment 1 replicate 2 and experiment 2; Figure S4: Flow cytometry gating strategy for MHC class I and MHC class II expression on B cells and macrophages; Figure S5: TCR spectratyping of splenocytes from MHC-matched lines at 21 dpi; Figure S6: Swiss-Model predicted structures of line 6 and 7 TCR Vbeta-1 haplotypes; Figure S7: 3′ TCR Vbeta sequences in Illumina data; Figure S8: 3′ TCR Vbeta-1 codon usage in line 6 × 7 F1 RNA-seq data; Figure S9: De novo assembly of line 6 and line 7 Pacbio DNA-seq in Canu; Figure S10: Line 6 and line 7 TCR Vbeta-1 sequences from PacBio assemblies; Figure S11: Line 6 and Line 7 TCR Vbeta-2 sequences from Pacbio assemblies; Figure S12: Putative Line 6 and Line 7 TCR Vbeta-3 sequences from PacBio assemblies; Figure S13: UAO4 cell reactivation assays; Figure S14: In silico modeling of reference and variant TCRβ; Table S1: Proliferation assay sample sizes at each timepoint; Table S2: Vbeta-1 CDR3-adjacent search primers. Supplementary Data File S1: Illumina sequencing-derived DNA haplotypes for TCRVbeta-1 in Line 6 and Line 7.
