# Supplementary material for: Contribution of the TCRβ Repertoire to Marek’s Disease Genetic Resistance in the Chicken
Source: Viruses. 2023 Feb 22;15(3):607. doi: 10.3390/v15030607 (PMC10055752; doi:10.3390/v15030607)
Supplement: Supplementary file 1 [file viruses-15-00607-s001.zip › viruses-2070964-supplementary/Supplementary Figures.pdf]

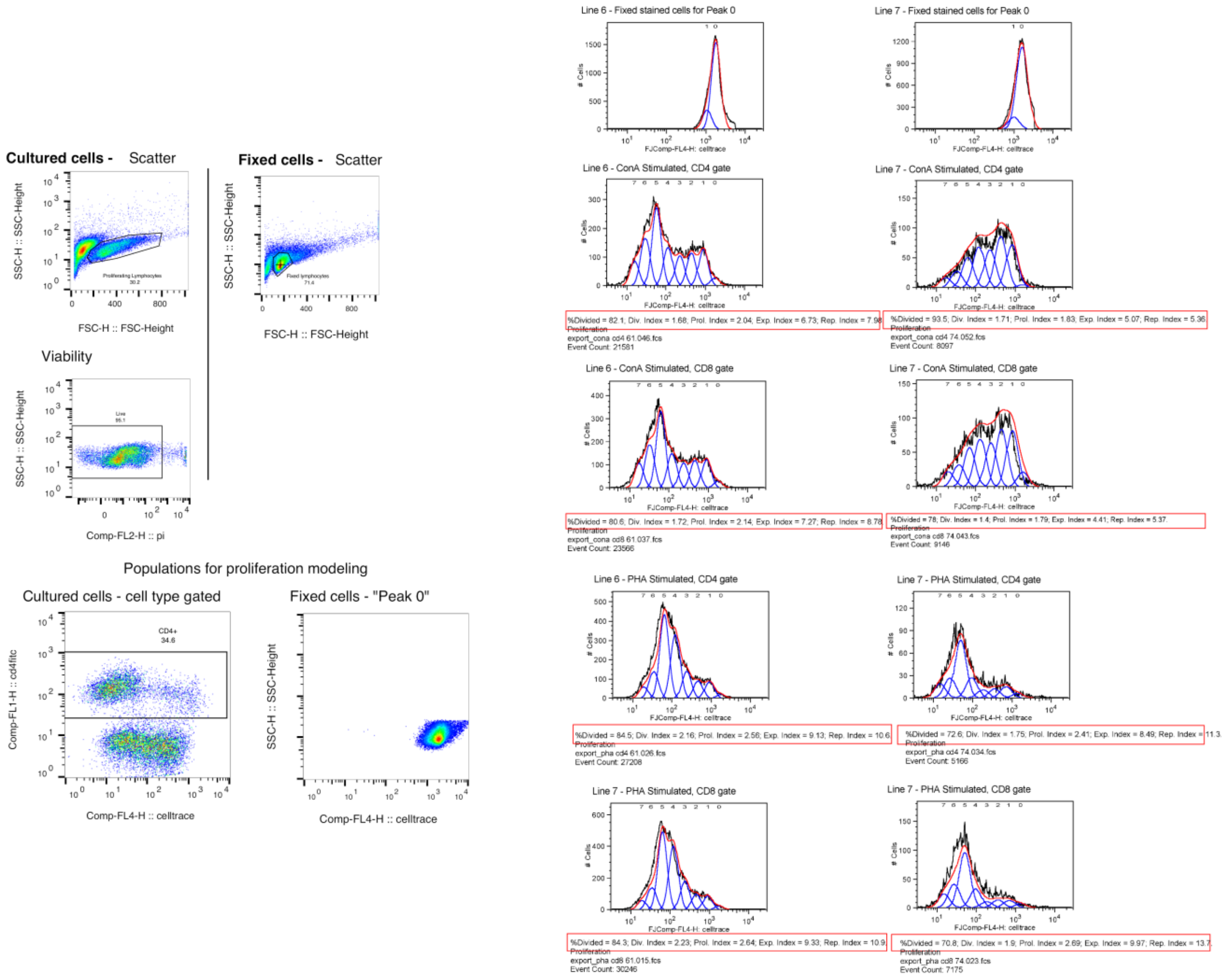


(**A**) (**B**)

**Figure S1.** Proliferation assay gating and modeling. (**A**). Proliferation assay cell gating. Flow cytometry was performed on Cell Trace Far Red-labeled, mitogen-proliferated samples from culture, as well as matched fixed Cell Trace-labeled samples to determine initial fluorescence. Cells were gated on lymphoblasts (cultured samples) or bulk lymphocytes (fixed cells). Cultured samples were gated on viability (either propidium iodide or Sytox Blue). Cultured samples were stained with either CD4-FITC or CD8-FITC, allowing population gating of CD4 and CD8 samples prior to proliferation modeling. (**B**). Proliferation modeling. Spleen samples were labeled with Cell Trace Far Red and proliferated in culture with either PHA or ConA. Gated viable CD4+ or CD8+ cells were modeled for proliferation in FlowJo V. 9, using matched fixed samples to determine peak 0 fluorescence. Proliferation index measured as total fold expansion of the gated population ("Expansion index" in FlowJo) is reported, with more proliferated samples having a higher fold expansion. N.B.: FlowJo reports a different metric than other platforms as "proliferation index" [49].

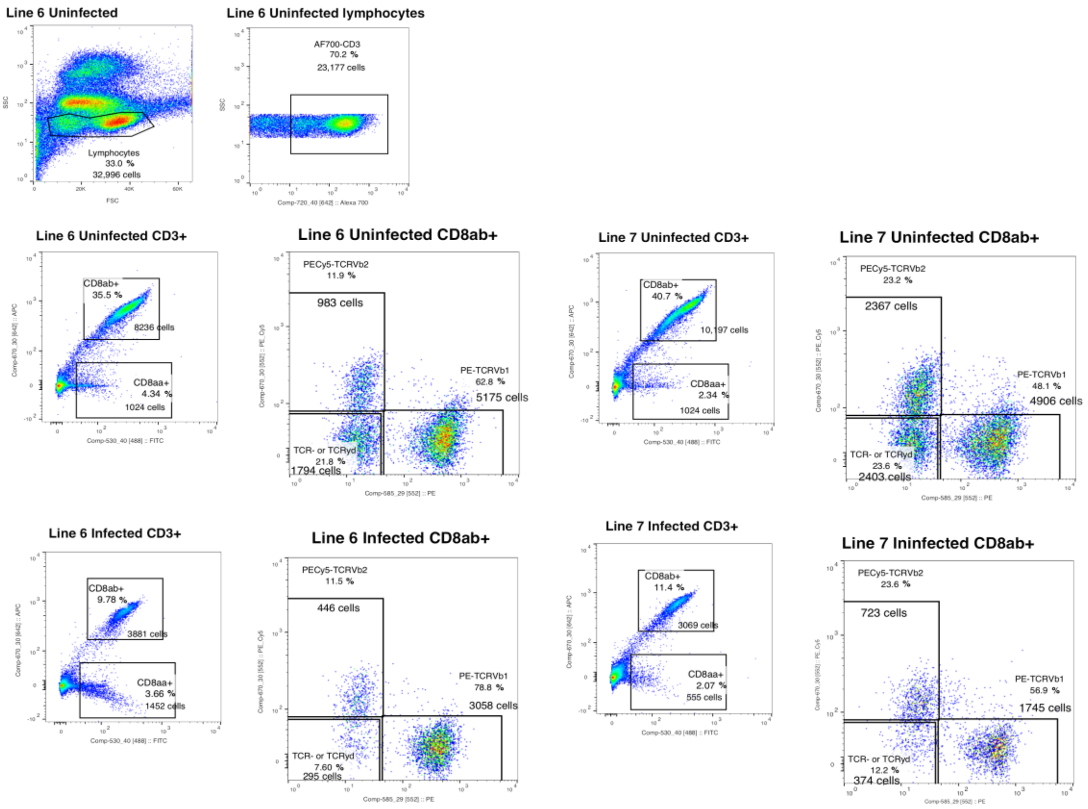


**Figure S2.** Flow cytometry gating strategy for Experiment 1 replicate 1. Fresh spleen samples were labelled for CD3-AF700, CD8alpha-FITC CD8beta-APC, TCR2-PE, and TCR3-PECy5. (**A**). Mononuclear cells were gated on FSC vs SSC, followed by gating of CD3+ cells. (**B**). CD8alphabeta+ cells were subgated for TCR analysis. CD8aa+ cells were rare which may reflect antibody competition against the CD8aa homodimer in this assay. One bird each is shown from line 6 and line 7 uninfected and infected groups at day 21.

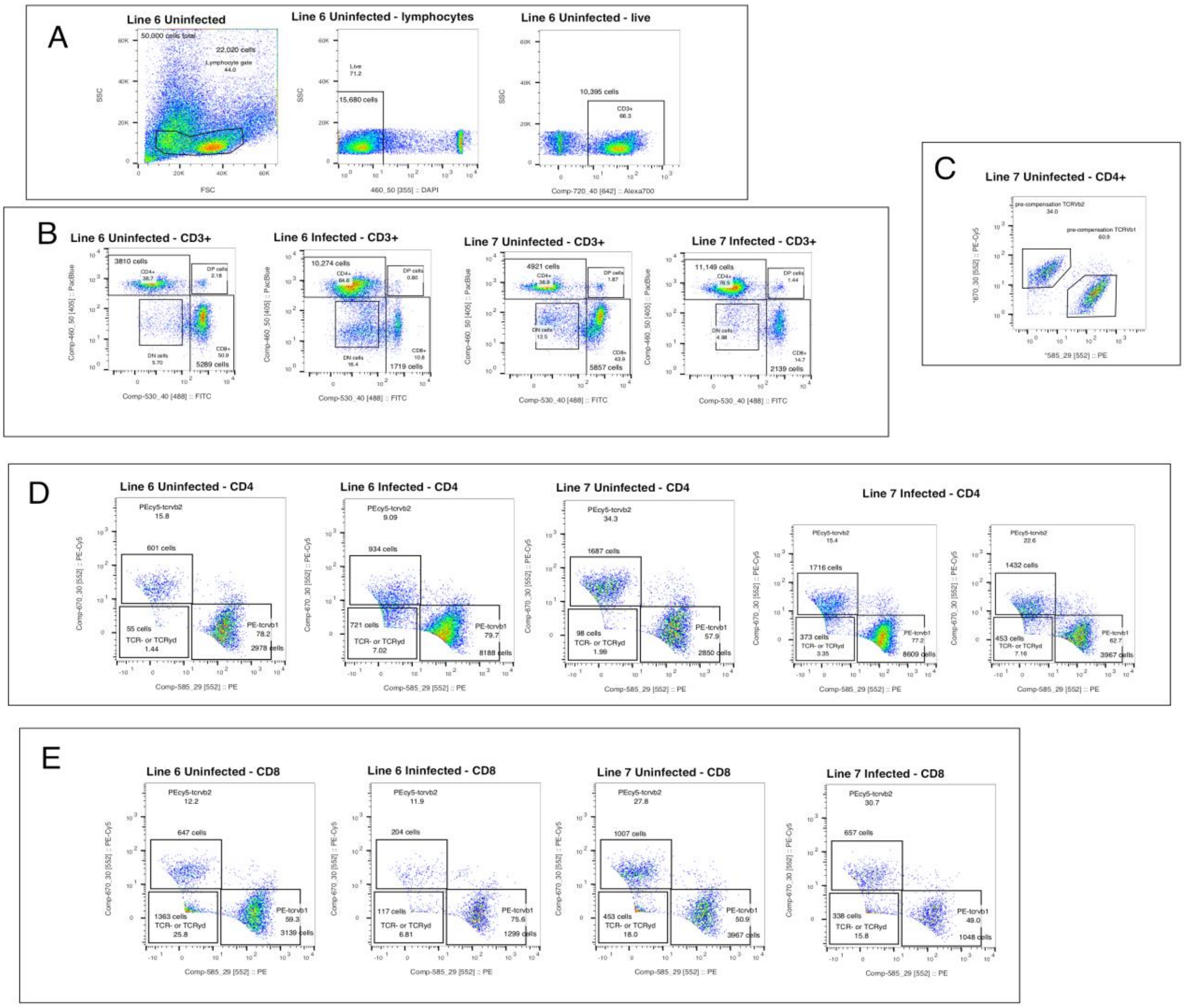


**Figure S3.** Flow cytometry gating strategy for Experiment 1 replicate 2 and Experiment 2. Data from Experiment 1 replicate 2, day 21 post-infection is shown. Fresh spleen samples from birds infected with MDV or unchallenged were labelled with antibodies against CD3-AF700, CD4-Pacific Blue, CD8alpha-FITC, TCR2-PE, and TCR3-PECy5, and viability-stained with DAPI. (**A**). Mononuclear cells were gated on FSC vs SSC, followed by stringent viability gating on uncompensated DAPI, and then gating on CD3+ cells. (**B**). CD3+ cells were subgated for CD4 and CD8; one sample each is shown for lines 6 and 7, uninfected and infected. (**C**). Machine optimization placed negative populations on the axis for TCR markers, but positive populations were well-separated (uncompensated sample shown for comparison to compensated data). (**D**-**E**). TCR2+ vs TCR3+ expression are shown for CD4+ cells (Subfigure D) and CD8+ cells (Subfigure E). One bird is shown for each line, infection condition, and cell type in Subfigures D and E, except for Line 7 Infected-CD4 cells of which two birds are shown representative of the high variability seen in this group.

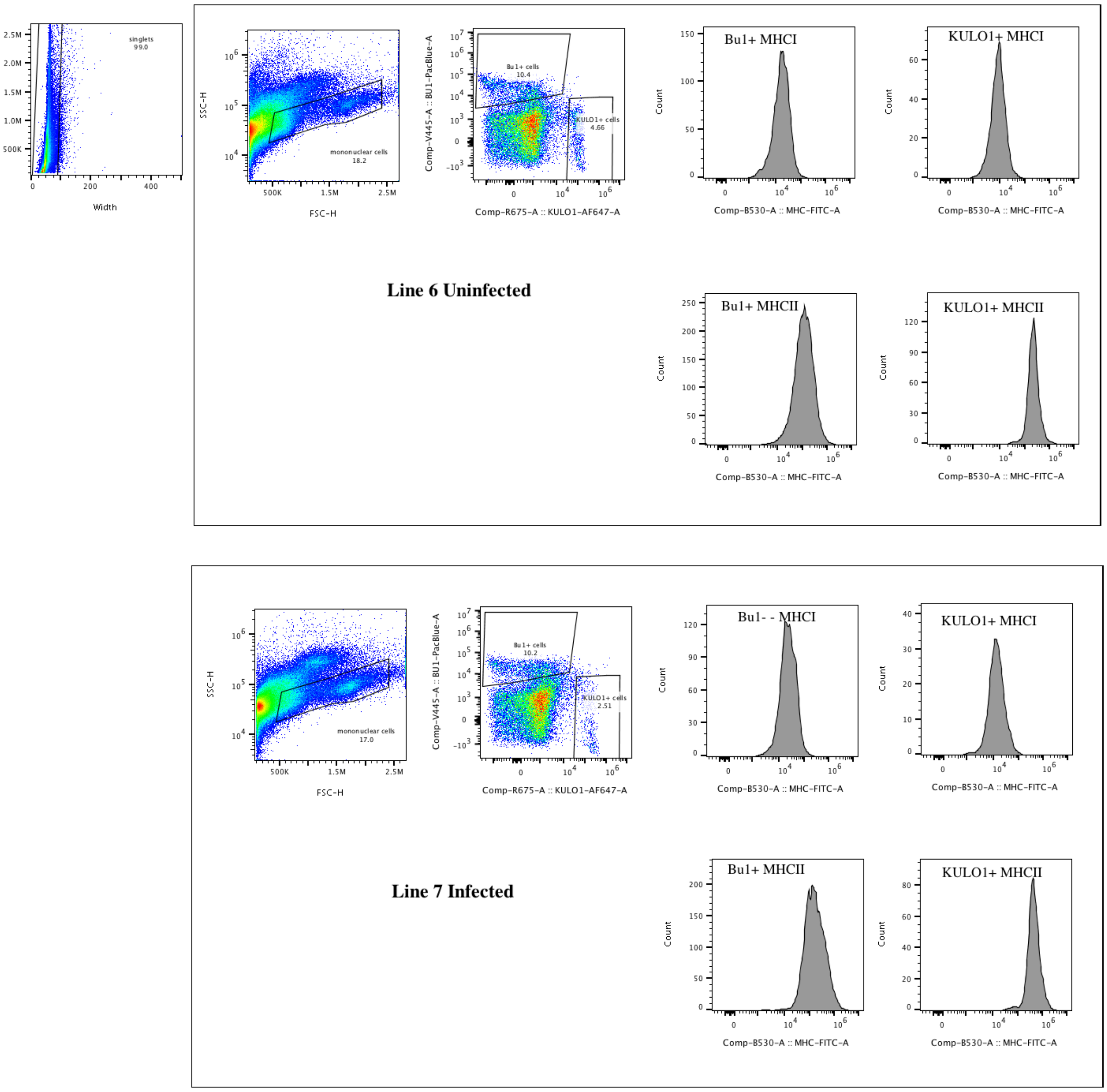


**Figure S4.** Flow cytometry gating strategy for MHC class I and MHC class II expression on B cells and macrophages. Cryopreserved spleen samples from MD-infected and control birds were thawed for less than 1 minute at 95 degrees C to minimize macrophage lysis. Samples were immunolabelled with Bu1-Pacific Blue, KUL01-AF647, and either MHC class I-FITC or MHC class II-FITC. One bird each is shown from the line 6 control group and line 7 challenged group. Gating was performed on FSC vs width for singlet analysis, FSC vs SSC for intact mononuclear cells, and Bu1+ and KUL01+ populations prior to estimation of median fluorescence intensity of MHC I-FITC or MHC II-FITC (separate sample aliquots were analyzed for each).

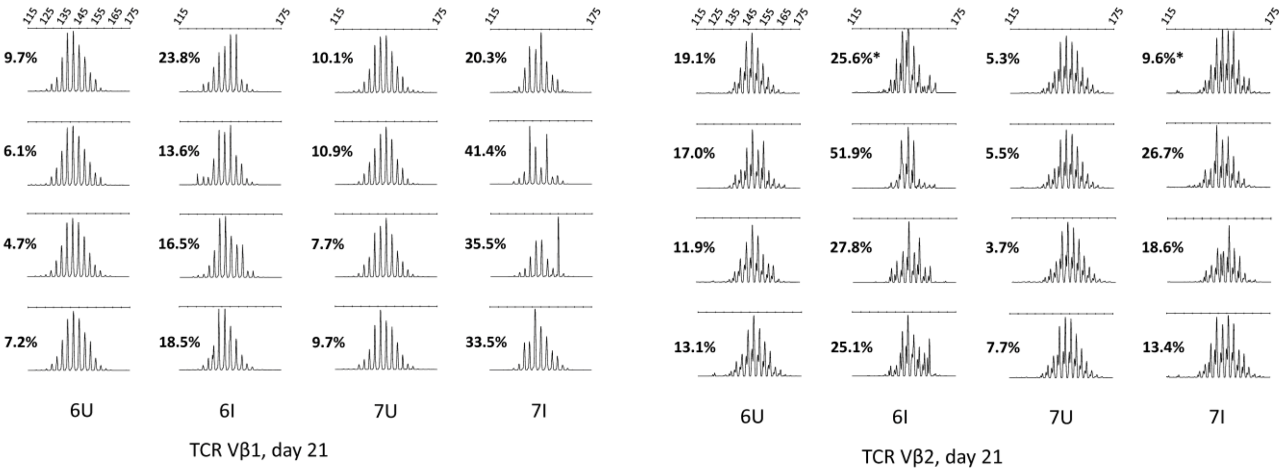


(**A**) (**B**)

**Figure S5.** TCR spectratyping of splenocytes from MHC-matched lines at 21 dpi. Divergence scores are indicated as percentages, and horizontal scale axes indicate fragment size. (a.) TCR Vbeta-1 and (b.) Vbeta-2 PCR fragments at 21 days post-infection with MDV. Individual birds from Lines 6 and 7 are shown without (6U, 7U) or with (6I, 7I) MDV infection.

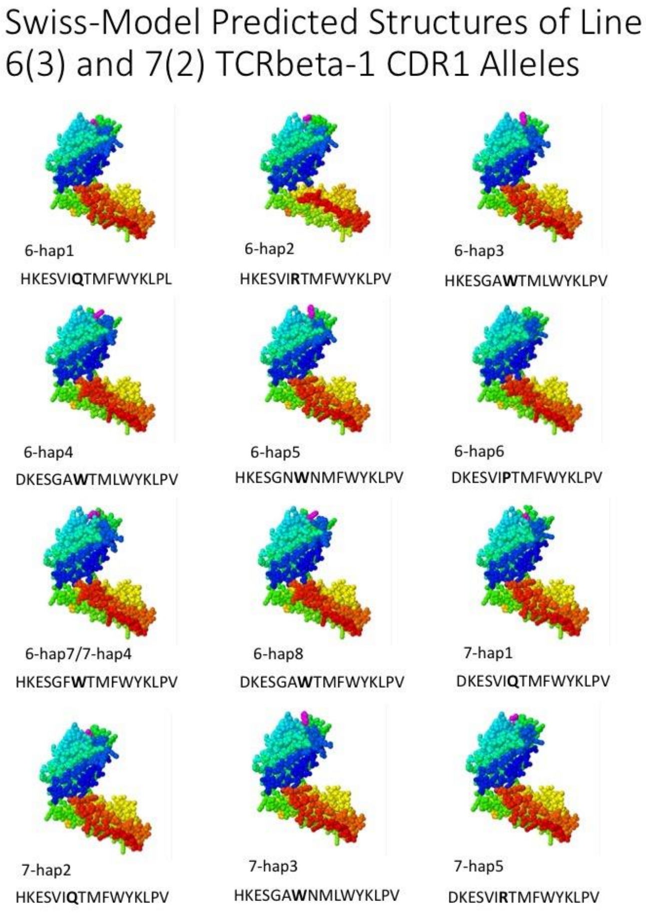


**Figure S6.** Swiss-Model predicted structures of line 6 and 7 TCR Vbeta-1 haplotypes. CDR1 Haplotypes identified in Illumina DNA-seq data for Lines 6 and 7. Models were built by incorporating the indicated CDR1 haplotypes into a reference TCR Vbeta-1 sequence and predicting each structure against a murine template, demonstrating that the hypervariable sequences fell within the

immunoglobulin domain B-C loop as expected. One haplotype (6-hap7/7-hap4) was shared between lines. CDR1 residue 44 is highlighted in fuchsia within each model, and indicated in bold within the CDR1 haplotype. Other residues in each model are colored by position along the protein chain using a reverse rainbow (bgyor) gradient.

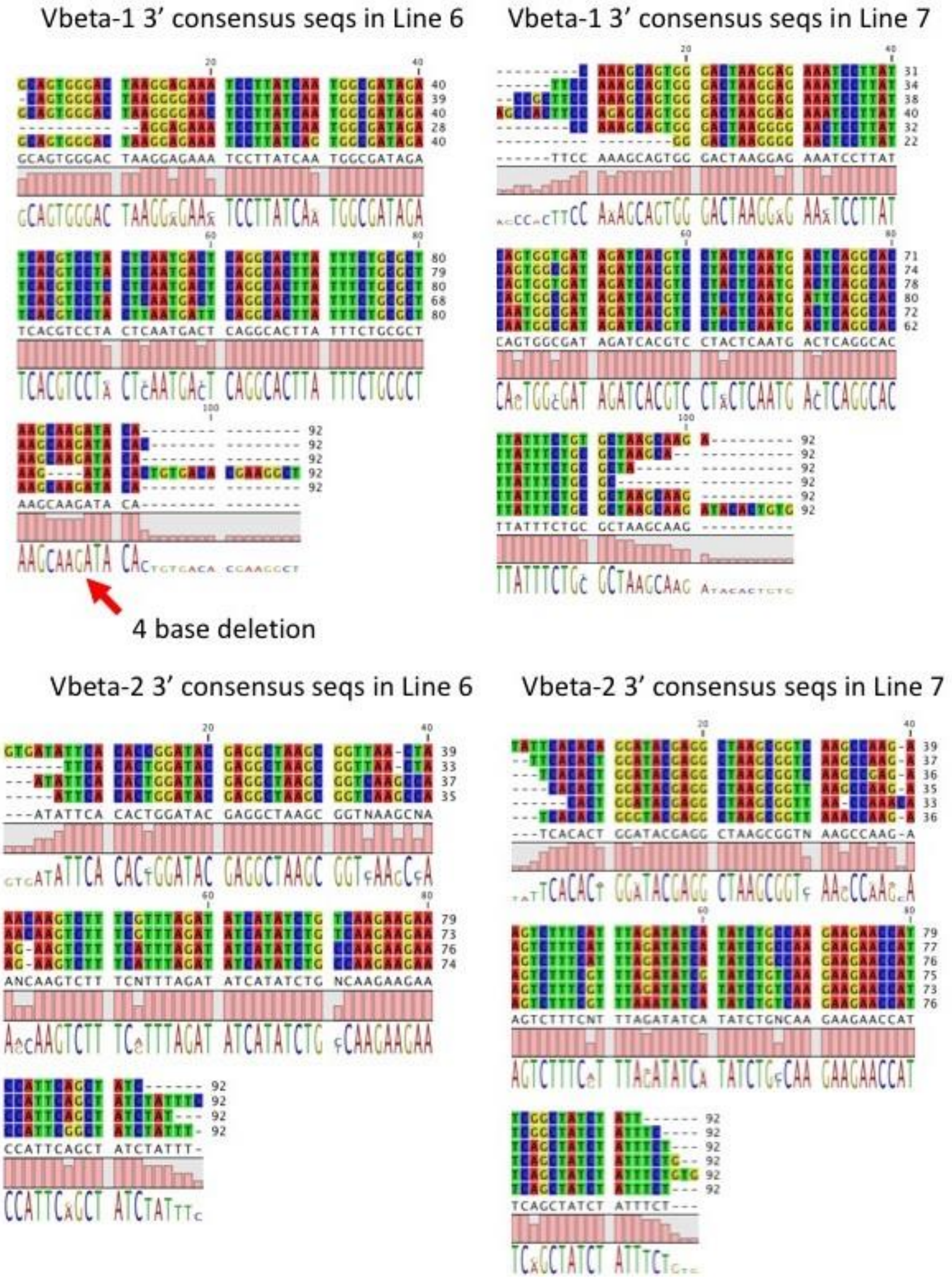


**Figure S7.** 3′ TCR Vbeta sequences in Illumina data. A: Illumina DNA-seq data from line 6 and line 7 birds were interrogated for conserved Vbeta-1 and Vbeta-2 sequences upstream of the 3′ RSS using the search primers listed in Supplementary Table 2, and consensus sequences aligned. A 4-base deletion upstream of the RSS was identified in one line 6 Vbeta-1 sequence; no evidence for this deletion was found in individual read data of line 7. $n$ = 1 pool of 7 birds per line. Alignments were performed and analyzed in CLC Genomics Workbench v. 7.5.1 (Qiagen, Hilden, Germany).

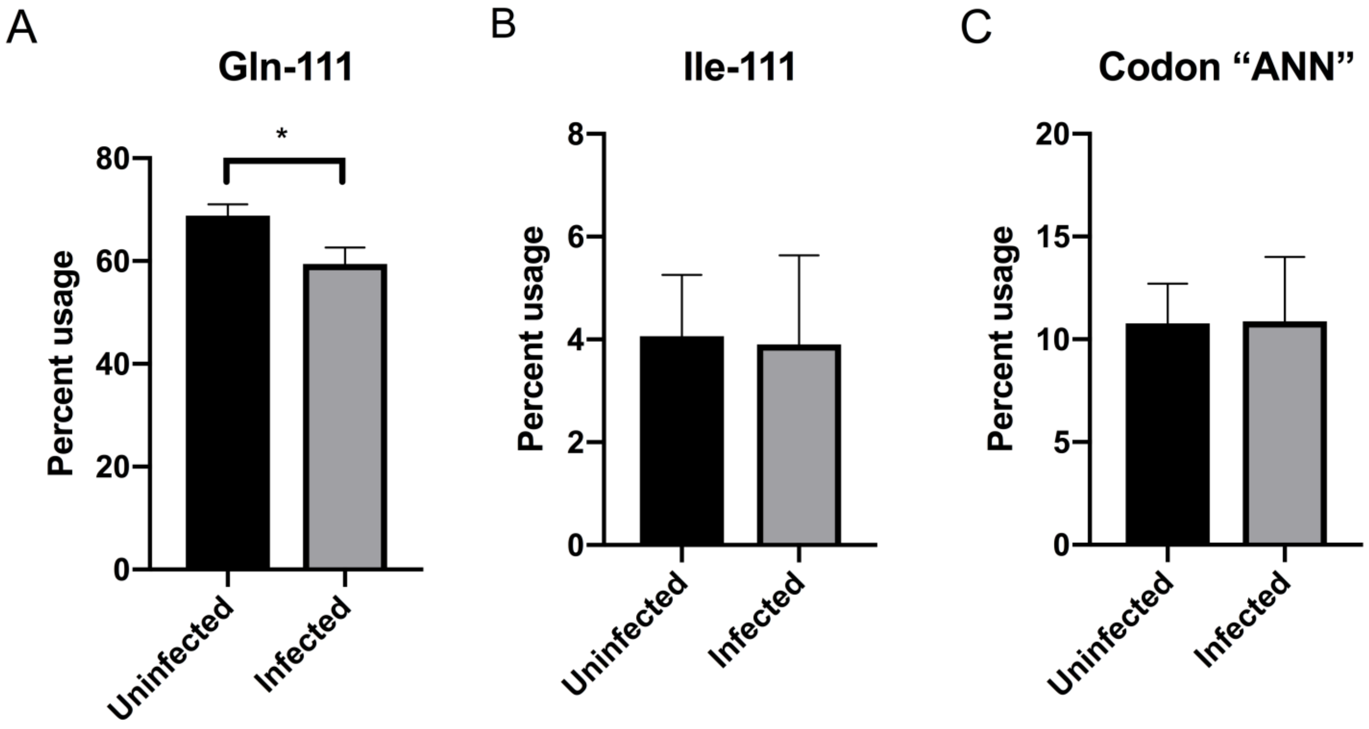


**Figure S8.** 3′ TCR Vbeta-1 codon usage in line 6x7 F1 RNA-seq data. Illumina RNA-seq was performed on splenocytes from uninfected birds or birds infected at 4 dpi with MDV. RNA-seq reads containing the search primers in Supplementary Table S2 were interrogated for use of A: canonical (Gln-111), B: non-canonical (Ile-111), or C: adenine-initiated ('ANN') codons at the Vbeta-1-CDR3 junction in reads with canonical sequences through codon 110. * = $p < 0.05$ by Student's T test. $n = 3$ birds analyzed per group, with 164, 131, and 220 Vbeta-1-CDR3-containing reads with canonical codon 110 usage identified in the uninfected samples, and 403, 111, and 164 in the infected samples.

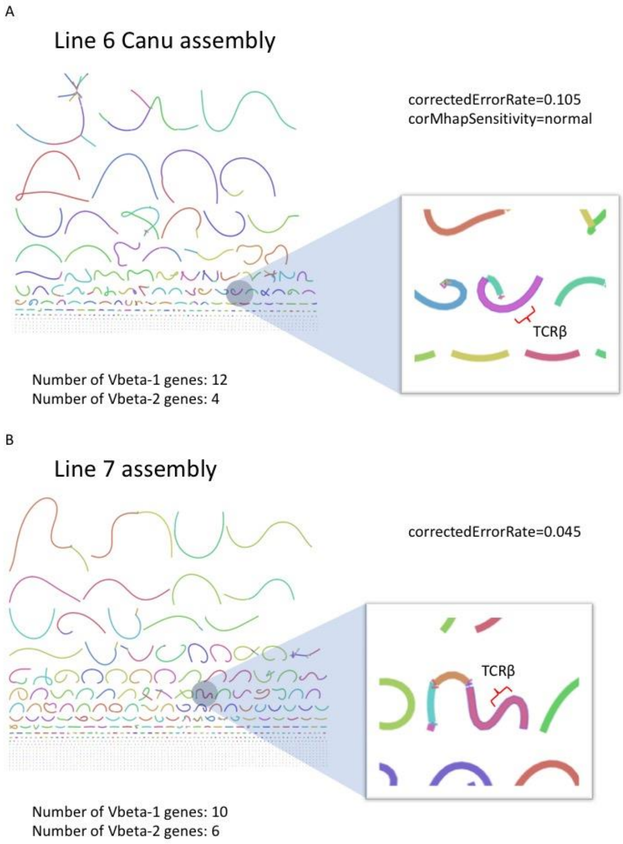


**Figure S9.** De novo assembly of line 6 and line 7 Pacbio DNA-seq in Canu. TCR beta locus assembly was examined for contiguity in Bandage. (**A**): Relaxed stringency was required to assemble the complete Line 6 TCR beta locus on one contig. (**B**): Assembly with default stringency parameters produced a contiguous TCR beta locus. Stringency parameters used and number of TCR variable genes resolved by each assembly are indicated.

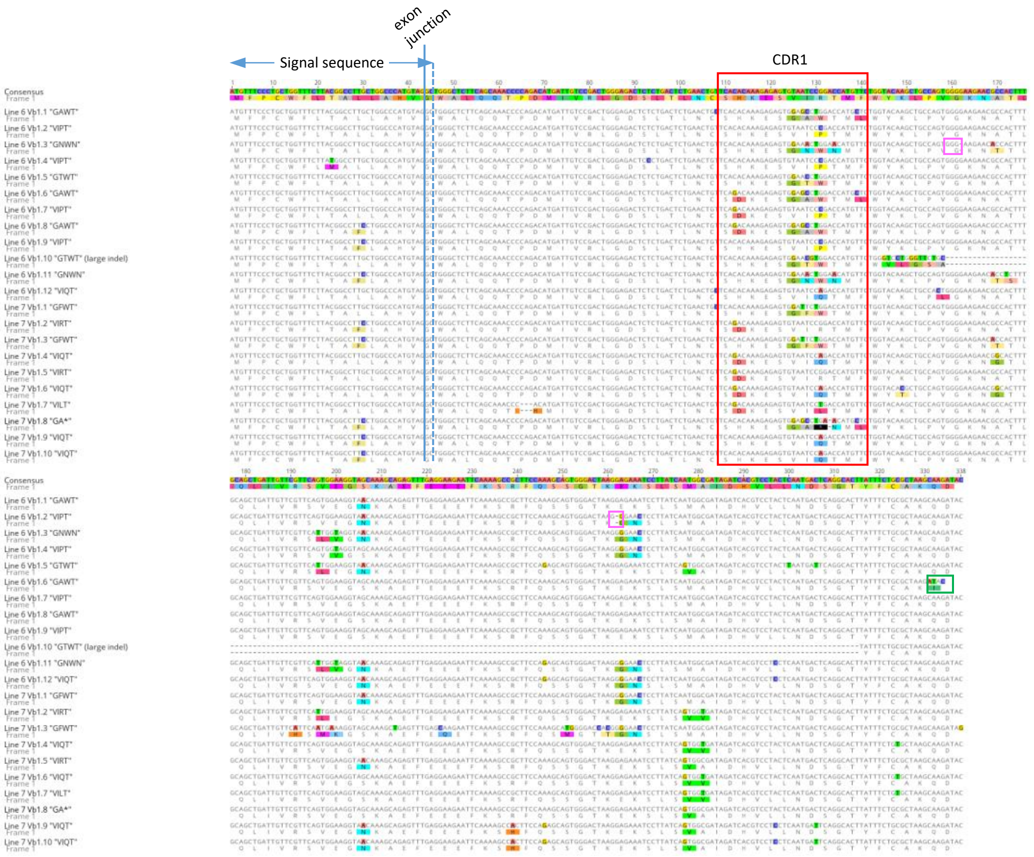


**Figure S10.** Line 6 and Line 7 TCR Vbeta-1 sequences from PacBio assemblies. Aligned coding sequences; CDR1 region is highlighted in red. Two single-base deletions in homoguanine tracts consistent with PacBio sequencing errors are outlined in pink. Site of 4-base deletion resulting in 111Q->111I is outlined in green. Translation relative to consensus sequence (ignoring the possible sequencing-related indels) is shown.

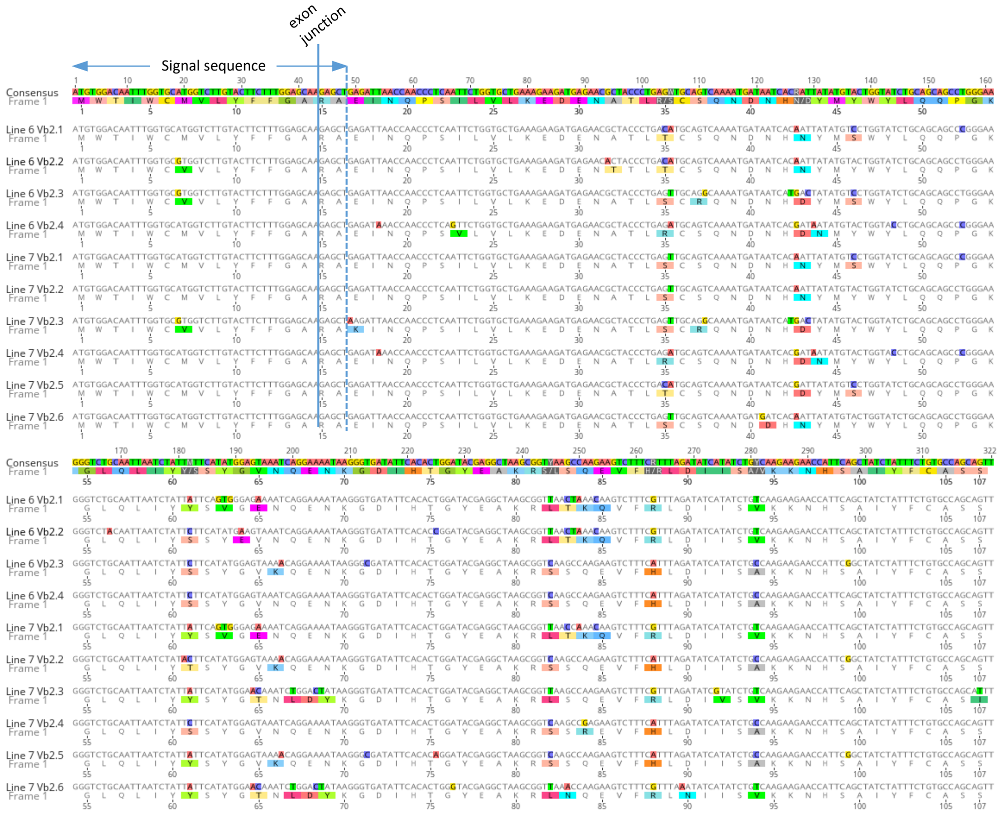


**Figure S11.** Line 6 and Line 7 TCR Vbeta-2 sequences from PacBio assemblies. Aligned coding sequences. Translation relative to consensus sequence is shown.

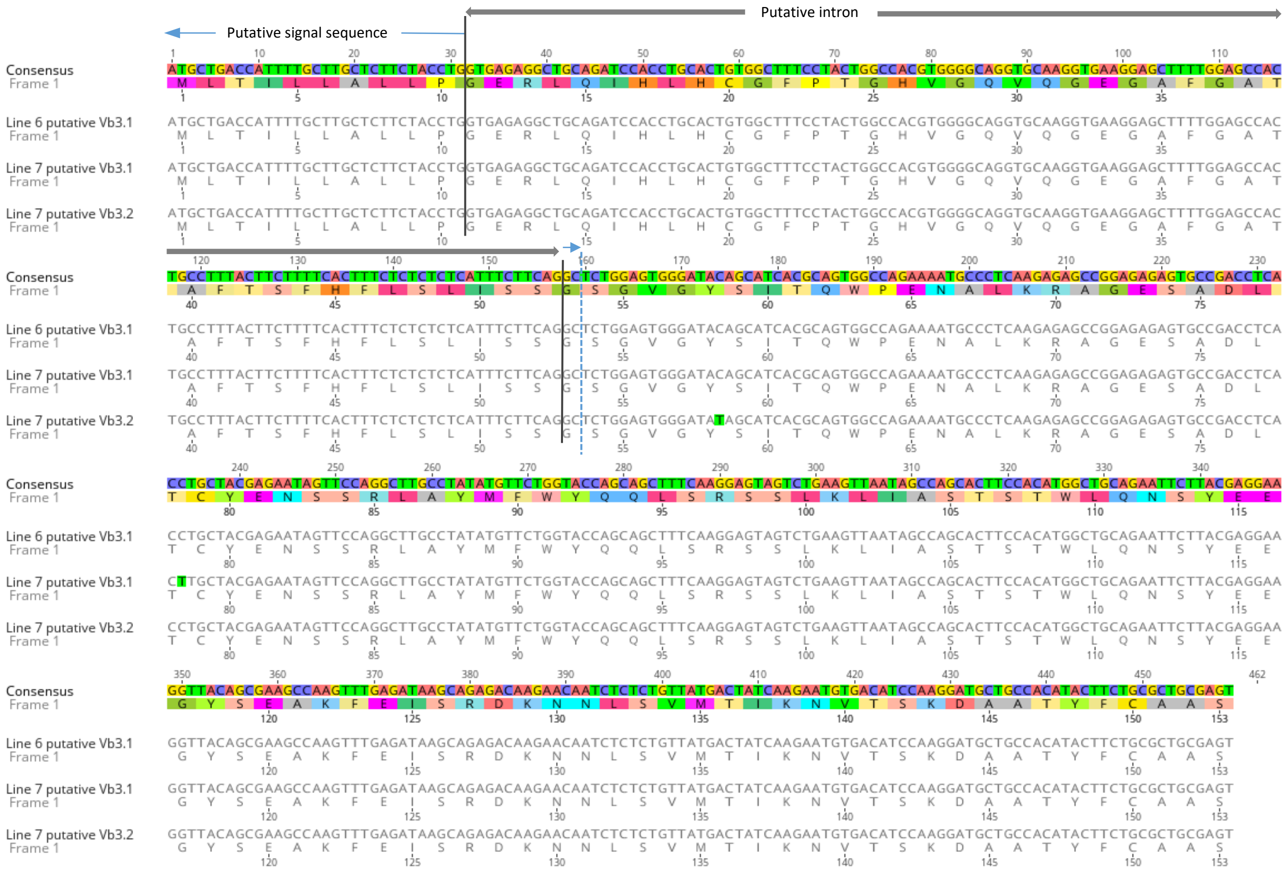

**Figure S12.** Putative Line 6 and Line 7 TCR Vbeta-3 sequences from PacBio assemblies. Aligned full sequences. Translation relative to consensus sequence is shown. Putative exon-intron junctions and signal sequence for Vbeta-3 derived from Zhang, et al., 2020 [45].

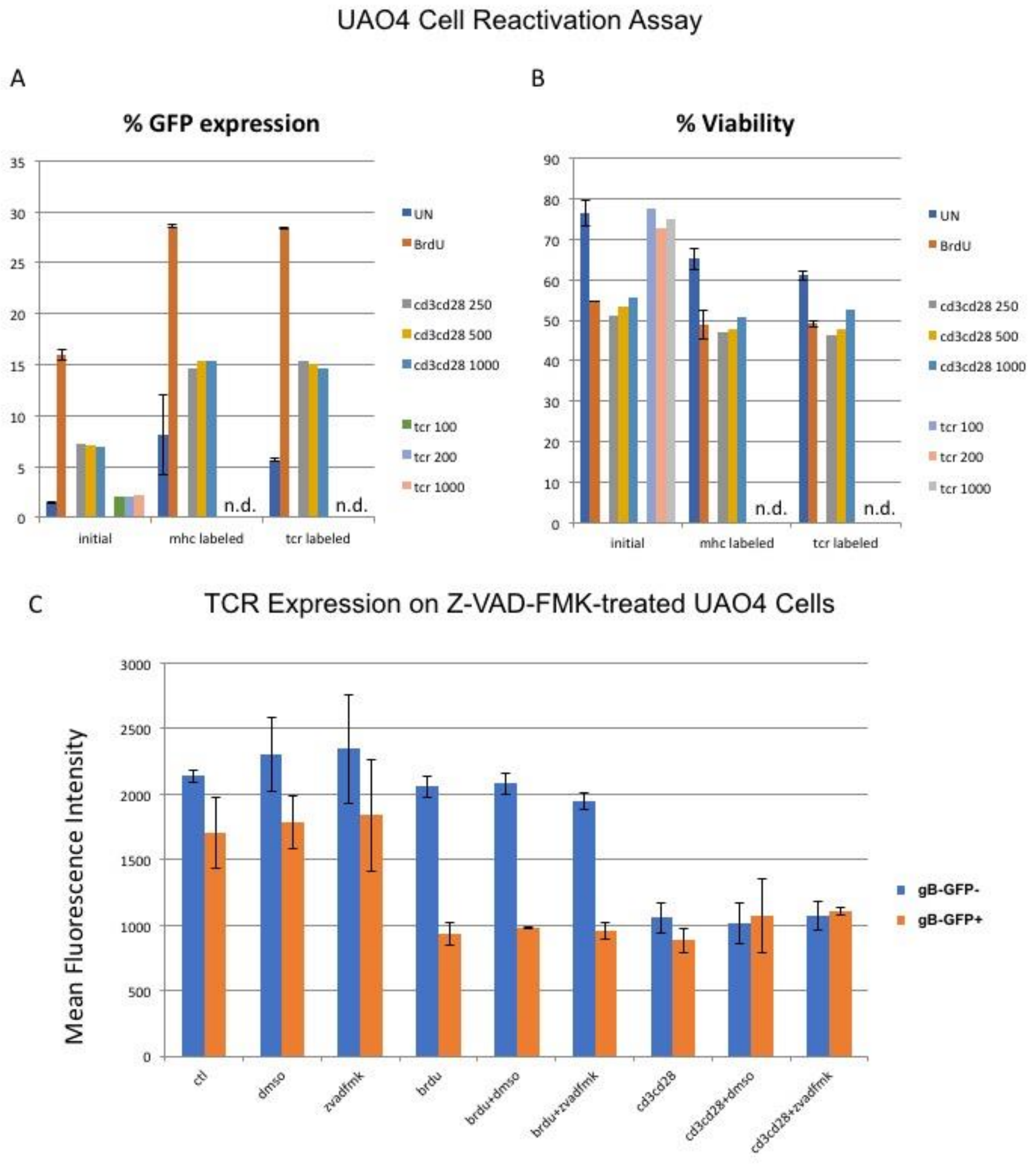


**Figure S13.** UAO4 cell reactivation assays. A: Stimulation with BrdU and CD3/CD28 antibodies, but not anti-TCR antibody alone, reactivates MDV (measured as gB-GFP expression) in latently infected UAO4 cells. MHC and TCR labeling independently mildly increased gB-GFP expression. Response to CD3/CD28 antibodies was not concentration-dependent across 3 dilutions (1:250, 1:500, 1:1000), therefore these dilutions were treated as replicates for analysis of TCR and MHC II expression. B: Viability was equivalently reduced to ~50% by reactivation with either BrdU or CD3/CD28 antibodies. $n$ = 3 for unstained and BrdU treated samples; $n$ = 1 for individual concentrations of CD3/CD28 or anti-TCR antibody. n.d. = not done. C: Caspase inhibition reactivation assay. Broad-acting caspase inhibitor Z-VAD-FMK did not rescue TCR expression in reactivating UAO4 cells. Cells were treated with 10uM, 25uM, and 50uM of Z-VAD-FMK or an equivalent volume of DMSO and reactivated; average of Z-VAD-FMK titrations is shown, as TCR expression was not concentration-dependent. $n$ = 3 samples per assay condition.

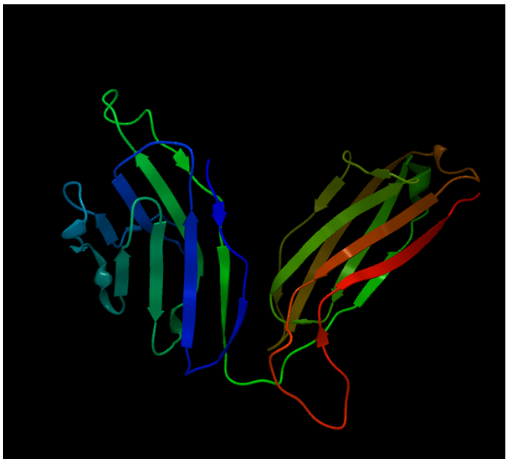

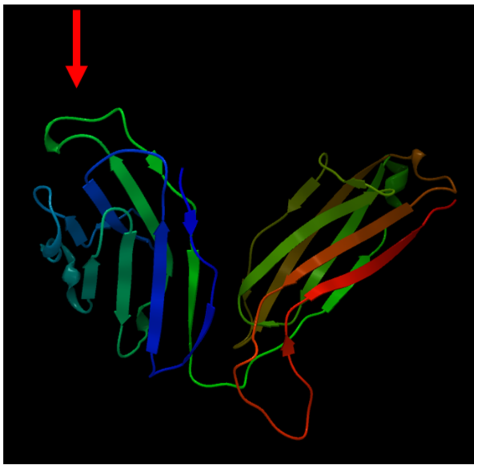

Gln-111 (Reference)    Ile-111 (Variant)

**Figure S14.** In silico modelling of reference and variant TCRβ. A reference chicken TCRβ containing Vβ1 (Genbank ABU93628.1) was modeled with default Swiss-Model parameters against a crystallography-supported mouse TCR-pMHC structure (PDB ID 3mbe.1.E). The chicken TCRβ formed the expected two-barrel structure. Substitution of Gln-111 with Ile-111 at the base of the CDR3 loop resulted in a predicted change in orientation of the CDR3 loop (red arrow).

**Table S1.** Proliferation assay sample sizes at each time point.

| Proliferation - ConA | Line 6 | Line 7 |
|---|---|---|
| Day 5 | *n* = 3 pools of 4 | *n* = 3 pools of 4 |
| Day 15 | *n* = 5 pools of 2 | *n* = 4 pools of 2 |
| Day 22 | *n* = 4 pools of 2 | *n* = 5 pools of 2 |
| Day 26 | *n* = 5 individuals | *n* = 5 individuals |
| Day 36 | *n* = 4 individuals | *n* = 5 individuals |
| **Proliferation - PHA** | | |
| Day 5 | (Not done) | (Not done) |
| Day 15 | *n* = 5 pools of 2 | *n* = 6 pools of 2 |
| Day 22 | *n* = 5 pools of 2 | *n* = 6 pools of 2 |
| Day 26 | *n* = 4 individuals | *n* = 4 individuals |
| Day 36 | *n* = 4 individuals | *n* = 3 individuals |

Spleens were pooled (days 5-22 post hatch) or assayed individually (days 26 and 36 post hatch), and depending on cell recovery after leucocyte enrichment and labeling, proliferation assays were performed for each sample with ConA, PHA, or both, to the sample sizes indicated.

**Table S2.** Vbeta-1 CDR3-adjacent search primers.

| Vbeta-1 Search Primers (Forward Direction) | Vbeta-1 Search Primers (Reverse-Complemented Direction) |
|---|---|
| AATGGTTCAGACACTTATTT | AAATAAGTGCCTGAGTCATT |
| AATGATTCAGACACTTATTT | AAATAAGTGCCTGAGCCATT |
| AATGGCTCAGACACTTATTT | AAATAAGTGCCTGAATCATT |
| AATGACTCAGACACTTATTT | AAATAAGTGCCTGAACCATT |
| AATGGTTCAGGCACTTATTT | AAATAAGTGTCTGAGTCATT |

| | |
|---|---|
| AATGATTCAGGCACTTATTT | AAATAAGTGTCTGAGCCATT |
| AATGGCTCAGGCACTTATTT | AAATAAGTGTCTGAATCATT |
| AATGACTCAGGCACTTATTT | AAATAAGTGTCTGAACCATT |

Search strings (primers) spanning TCR Vbeta-1 nucleotides 301-320, adjacent to the 5′ end of the CDR3 region, were used to identify CDR3-junctional Illumina RNAseq reads in Line 6x7 F1 spleen samples, using Fastq-grep [62]. These strings incorporated all variants (highlighted) identified across chicken TCR Vbeta-1 mRNAs uploaded in GenBank, and were searched in both the forward (column 1) direction and reverse-complemented (column 2) direction. Forward and reverse-complement searches were performed separately for each sample to allow identified reads to be converted to the forward orientation and aligned. Only two of the search string variants ("AATGA**C**TCAGGCACTTATTT" and "AATGA**T**TCAGGCACTTATTT", differing at nucleotide 306C/T) were found within the Line 6x7 F1 samples, except for a single read in one sample containing 305G.
